# Development of an R4 dual-site (R4DS) gateway cloning system enabling the efficient simultaneous cloning of two desired sets of promoters and open reading frames in a binary vector for plant research

**DOI:** 10.1371/journal.pone.0177889

**Published:** 2017-05-16

**Authors:** Mostafa Aboulela, Yuji Tanaka, Kohji Nishimura, Shoji Mano, Mikio Nishimura, Sumie Ishiguro, Tetsuya Kimura, Tsuyoshi Nakagawa

**Affiliations:** 1 Department of Molecular and Functional Genomics, Interdisciplinary Center for Science Research, Organization for Research, Shimane University, Matsue, Japan; 2 Bioresources Science, The United Graduate School of Agricultural Sciences, Tottori University, Tottori, Japan; 3 Department of Botany and Microbiology, Faculty of Science, Assiut University, Assiut, Egypt; 4 Department of Evolutionary Biology and Biodiversity, National Institute for Basic Biology, Okazaki, Japan; 5 Department of Basic Biology, School of Life Science, SOKENDAI (The Graduate University for Advanced Studies), Okazaki, Japan; 6 Department of Cell Biology, National Institute for Basic Biology, Okazaki, Japan; 7 Department of Biological Mechanisms and Functions, Graduate School of Bioagricultural Sciences, Nagoya University, Nagoya, Japan; 8 Department of Life Sciences, Graduate School of Bioresources, Mie University, Tsu, Japan; University College Dublin, IRELAND

## Abstract

Vast numbers of proteins work cooperatively to exert their functions in various cells. In order to understand the functions and molecular mechanisms of these proteins in plants, analyses of transgenic plants that concomitantly express two protein-coding genes are often required. We developed a novel Gateway cloning technology-compatible binary vector system, the R4 dual-site (R4DS) Gateway cloning system, which enables the easy and efficient cloning of two desired sets of promoters and open reading frames (ORFs) into a binary vector using promoter and ORF entry clones. In this system, C-terminal fusions with 17 kinds of tags including visible reporters and epitope tags are available for each ORF, and selection by four kinds of resistance markers is possible. We verified that the R4DS Gateway cloning system functioned well in *Arabidopsis thaliana* by observing the expression and localization patterns of fluorescent proteins fused with organelle-targeting signals and driven by stomatal-lineage specific promoters. We also confirmed that the two cloning sites in the R4DS Gateway cloning system were equivalent and independently regulated. The results obtained indicate that the R4DS Gateway cloning system facilitates detailed comparisons of the expression patterns of two promoters as well as co-localization and interaction analyses of two proteins in specific cells in plants.

## Introduction

With advances in omics databases and bioinformatics, many genes and their products have been anticipated to work cooperatively. In order to verify and utilize information on relationships between genes evaluated by these studies, transgenic analyses of expression patterns, intracellular dynamics, and molecular interactions between two genes of interest are necessary. Several approaches such as crossing, re-transformation, co-transformation, polycistronic transgenes, a polyprotein production system, bi-directional promoter strategy, and multi-gene construction are performed for the delivery of two or more genes into plant genomes [1, 2]. Successive rounds of crossing or sequential re-transformation are laborious and time-consuming. In re-transformation, different selection markers are required in each transformation process. Furthermore, transgenes are integrated at different loci and segregate in subsequent generations, which is also the case in co-transformation. Other methods use one T-DNA carrying all genes to be introduced for transformation and these genes are integrated at the same loci and inherited concurrently by offspring in successive generations. However, polycistronic transgenes, the polyprotein production system, and bi-directional strategy only use one promoter for the production of all encoded proteins [1, 3]. In contrast, multi-gene constructs carrying all expression cassettes in one T-DNA permit the use of different promoters in each expression cassette in order to independently regulate the expression of genes in each cassette [4, 5]. This characteristic is critically important because researchers need to carry out interactive functional analyses with multiple genes that are individually expressed using their own or suitable promoters (e.g. inducible, site-, or stage-specific promoters).

A binary vector system that provides researchers with the flexibility to choose the desired promoter(s) is expected to be useful practically because it is very laborious to construct several promoter-open reading frame (ORF) cassettes and arrange them in one binary vector using traditional cloning methods. Gateway cloning technology [6] has recently proved to be extremely useful for gene construction, and a number of Gateway cloning technology-compatible binary vectors for various applications including fusion with useful tags have been developed [7]. In addition to the originally utilized *att*1 and *att*2 sites, the engineered variants *att*3, *att*4, *att*5, and *att*6 were developed as new highly specific recombination sites and these made possible the multisite Gateway technology [8]. In the commercial multisite Gateway system, these six *att* sites (*att*1 to *att*6) are applied to the simultaneous linking and cloning of multiple DNA fragments (entry clones) into a vector by an LR reaction [9], for example, the linking of a promoter (*att*L1-promoter-*att*R5), ORF (*att*L5-ORF-*att*L4), tag (*att*R4-tag-*att*R3), and terminator (*att*L3-terminator-*att*L2) to make and clone expression cassettes (e.g. *att*B1-promoter-*att*B5-ORF-*att*B4-tag-*att*B3-terminator-*att*B2) [10]. Although this system is useful for assembling DNA fragments, it requires specialized entry clones, and the resources of universal entry clones accumulated in research communities are not available for this system. As alternative applications of multiple *att* sites, MultiRound Gateway technology [11, 12] and the Gateway recycling cloning system [13] have been developed for step-by-step repetitive cloning of an expression cassette into a vector to make a multi-gene binary construct using multiple rounds of LR reactions. Although these are outstanding methods to clone an unlimited number of expression cassettes into a binary vector, they require laborious traditional cloning steps to prepare a promoter:ORF construct on a prerequisite donor vector. Since many transgenic experiments require manipulations of up to two genes, the supportive binary vector system oriented simple cloning of just two expression cassettes is thought to be valuable as a practical tool.

In the present study, we developed a novel Gateway cloning technology-compatible binary vector system, the R4 dual-site (R4DS) Gateway cloning system to permit the easy and efficient cloning of two genes for various transgenic experiments in plants without the difficulties associated with other methods. In order to test this system, we made two-gene constructs with a combination of stomatal lineage-specific promoters and organelle-targeted fluorescent proteins, and observed consistent expression and localization patterns in transgenic *Arabidopsis thaliana*. This system provides a versatile cloning tool with multiple flexibility features, e.g., 17 types of C-terminal tags and 4 kinds of plant selection markers. The R4DS Gateway cloning system will be an invaluable experimental tool in plant research.

## Results and discussion

### Preparation of two-gene constructs and characteristics of the R4DS Gateway cloning system

**Fig 1:**
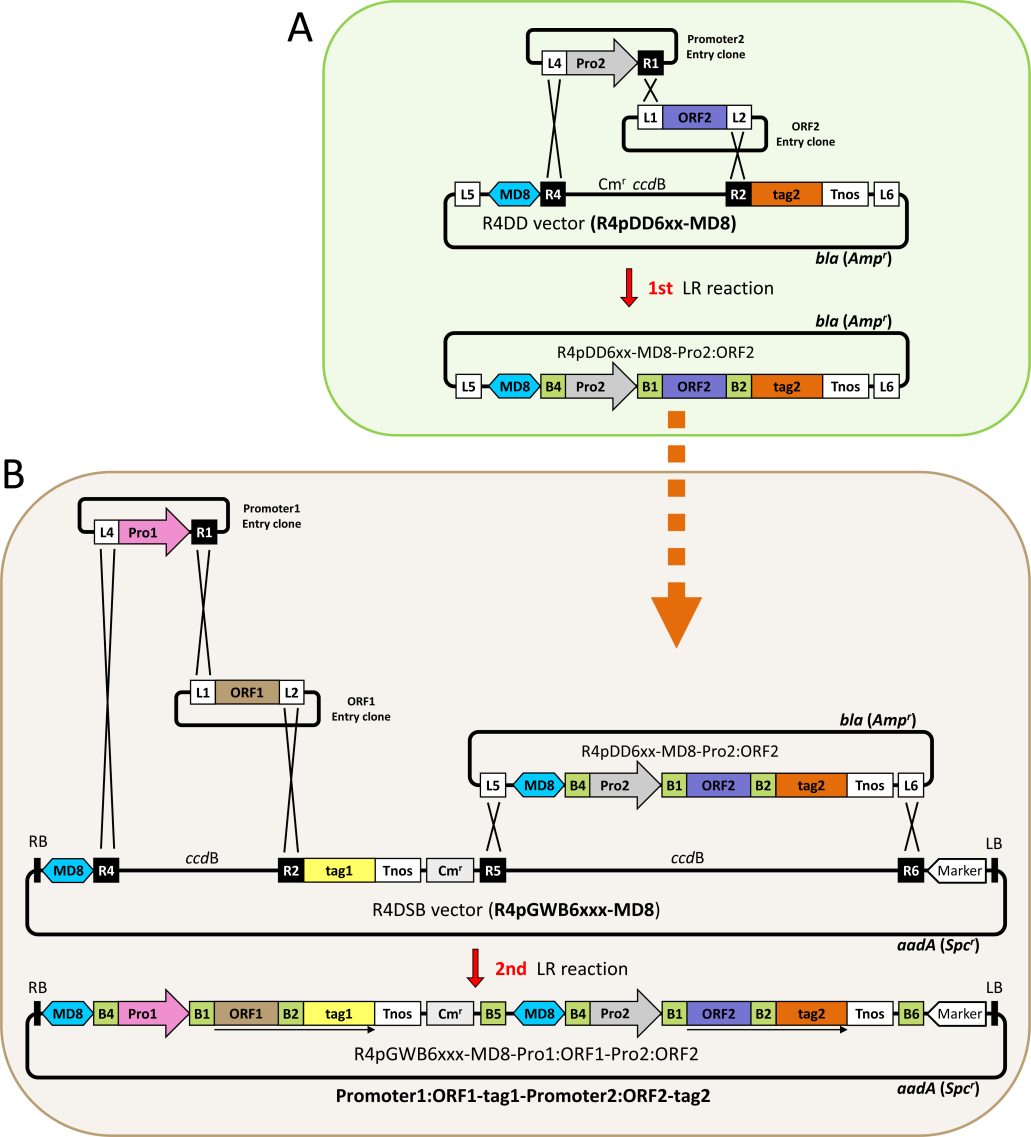
Outline for cloning two sets of promoters and ORFs in the R4DS Gateway cloning system. (A) The 1st LR reaction. In the 1st LR reaction (tripartite LR reaction), Pro2 and ORF2 are linked and cloned into an R4DD vector to make R4pDD6xx-MD8-Pro2:ORF2. The dashed arrow indicates that established R4pDD6xx-MD8-Pro2:ORF2 is used further for the 2nd LR reaction. (B) The 2nd LR reaction. In the 2nd LR reaction (quadripartite LR reaction), Pro1 and ORF1 are linked and cloned into an R4DSB vector together with the Pro2:ORF2-tag2 constructed by the 1st LR reaction to make the binary clone R4pGWB6xxx-MD8-Pro1:ORF1-Pro2:ORF2 with two independent expression cassettes. MD8, the matrix attachment region; B1, *att*B1; B2, *att*B2; B4, *att*B4; B5, *att*B5; B6, *att*B6; L1, *att*L1; L2, *att*L2; L4, *att*L4; L5, *att*L5; L6, *att*L6; R1, *att*R1; R2, *att*R2; R4, *att*R4; R5, *att*R5; R6, *att*R6; *ccd*B, Control of Cell Death B as a negative selection marker for bacteria; Cm^r^, chloramphenicol resistance; Marker, plant selection marker; Pro1, promoter1; Pro2, promoter2; LB, left border; RB, right border; Tnos, nopaline synthase terminator; *aadA*, the gene for spectinomycin resistance (Spc^r^) in bacteria; *bla*, gene for ampicillin resistance (Amp^r^) in bacteria. Arrows under ORF1-B2-tag1 and ORF2-B2-tag2 indicate expression. Figures are not drawn to scale.

**Fig 2:**
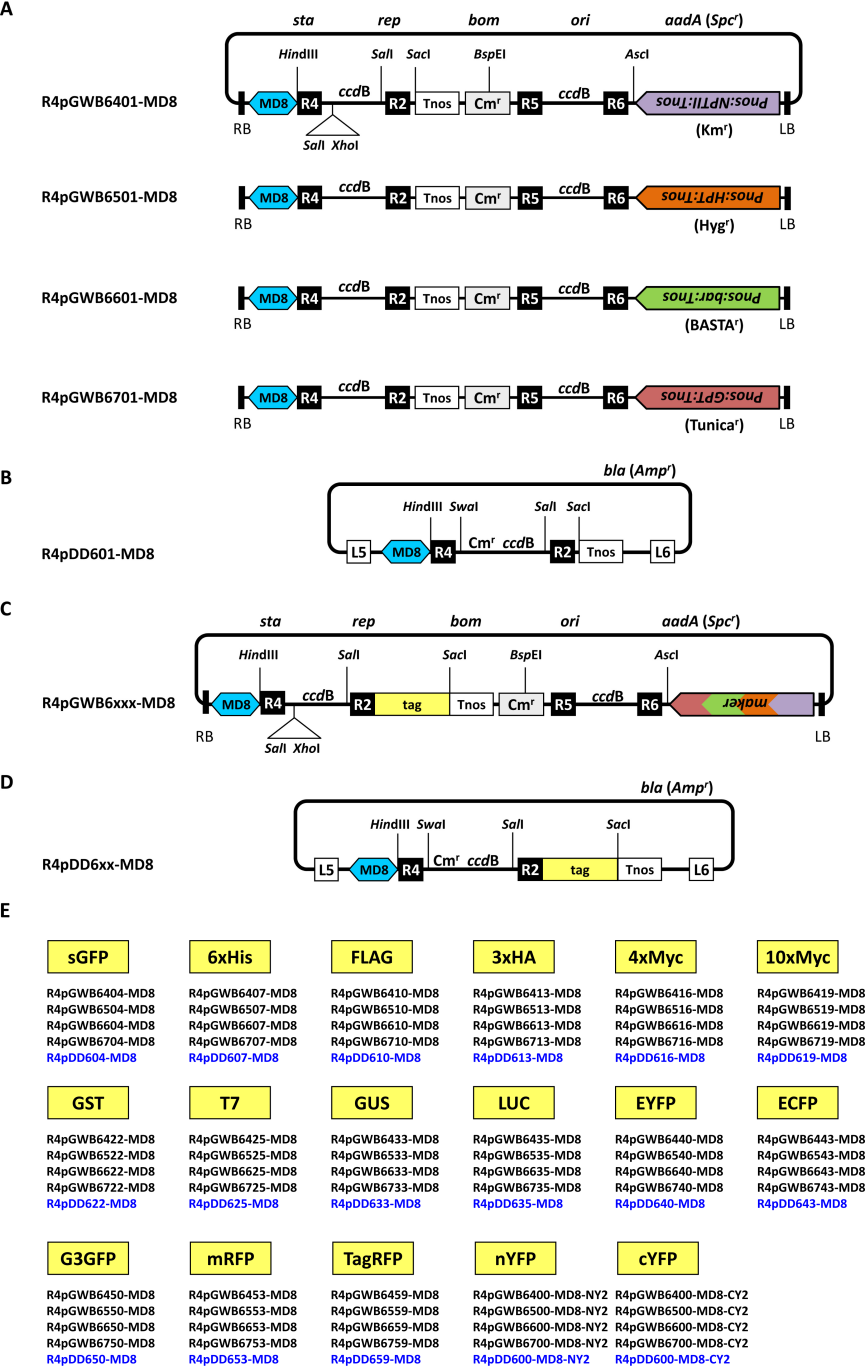
Line-up of R4DSB vectors (R4pGWB6xxx-MD8) and R4DD vectors (R4pDD6xx-MD8). (A) Structural diagrams of the four no tag-type R4DSB vectors: R4pGWB6401-MD8, R4pGWB6501-MD8, R4pGWB6601-MD8, and R4pGWB6701-MD8. Backbone and restriction sites were shown in R4pGWB6401-MD8. The only difference between these four vectors is the selection marker for plants. LB, left border; RB, right border; *sta*, the region conferring stability in *Agrobacterium tumefaciens*; *rep*, broad host range replication origin; *bom*, cis-acting element for conjugational transfer; *ori*, ColE1 replication origin; Pnos, nopaline synthase promoter; Tnos, nopaline synthase terminator. (B) Structural diagram of the no tag-type R4DD vector, R4pDD601-MD8. (C) Structural diagram of tag fusion-type R4DSB vectors (R4pGWB6xxx-MD8). These vectors have the same structure as the no tag-type vector R4pGWB6x01 represented in A, except for a tag downstream of *att*R2. (D) Structural diagram of tag fusion-type R4DD vectors (R4pDD6xx-MD8). These vectors have the same structure as no tag-type R4pDD601-MD8 represented in B, except for a tag downstream of *att*R2. (E) Tags carried in R4pGWB6xxx-MD8 and R4pDD6xx-MD8. Figures in A-D are not drawn to scale.

We herein developed the R4DS Gateway cloning system, which permits the easy and fast cloning of two sets of promoters and ORFs in a binary vector for plant transformation using any combination of a promoter entry clone (*att*L4-promoter-*att*R1) and ORF entry clone (*att*L1-ORF-*att*L2). ORFs may be fused with various tags (visible reporters or epitope tags; see below), if necessary. Fig 1 shows vector structures and the cloning procedure in this system. The system consists of two types of vectors, R4 destination donor (R4DD) vectors and R4 dual-site binary (R4DSB) vectors. We placed MD8 [14], a matrix attachment region (MAR) of *A*. *thaliana*, upstream of the expression cassettes of both vectors. MARs are expected to increase the overall levels of transgene expression and reduce variance in expression patterns in transgenic plants [15–20]. Two LR reactions are used to generate the two-gene constructs. The first LR reaction (tripartite LR reaction) connects promoter2 (Pro2) and ORF2 with tag2 in the R4DD vector and produces an intermediate clone (*att*L5-MD8-*att*B4-Pro2-*att*B1-ORF2-*att*B2-tag2-Tnos-*att*L6) [R4pDD6xx-MD8-Pro2:ORF2 in Fig 1A]. Tnos represents the terminator region of the nopaline synthase gene. The second LR reaction (quadripartite LR reaction) connects the second set of the promoter [promoter1 (Pro1)] and ORF entry clones (*att*L4-Pro1-*att*R1 and *att*L1-ORF1-*att*L2) and incorporates the two genes into R4pGWB6xxx-MD8 (Fig 1B). A final binary clone, MD8-*att*B4-Pro1-*att*B1-ORF1-*att*B2-tag1-Tnos-Cm^r^-*att*B5-MD8-*att*B4-Pro2-*att*B1-ORF2-*att*B2-tag2-Tnos-*att*B6 can be transferred into the plant genome using Agrobacterium-mediated transformation. In fusion-type vectors, the *att*B2 linker sequence between ORF and tag encodes 12 amino acids. Whereas, 13 additional amino acids representing the *att*B2 linker sequence are added at the C-terminus of the protein of interest in the case of vectors without a tag. For a detailed description of linker sequences, see Fig 2 and S1 Fig of Nakagawa *et al* (2008) [21].

The R4DS Gateway cloning system consists of four selection marker series (Fig 2). R4DSB vectors with the MD8 sequence were named R4pGWB6xxx-MD8 (Fig 2A and 2C). The first digit “6” of the four-digit number indicates the dual-site type (6xxx, vectors using *att*6 for cloning) and the next digit (4, 5, 6, or 7) refers to the types of plant selection markers. The R4pGWB64xx-MD8 series contains neomycin phosphotransferase II (NPTII) conferring kanamycin resistance (Km^r^), the R4pGWB65xx-MD8 series contains hygromycin phosphotransferase (HPT) conferring hygromycin resistance (Hyg^r^), the R4pGWB66xx-MD8 series contains phosphinothricin acetyl transferase (the bialophos resistance gene; bar) providing BASTA resistance (BASTA^r^), and the R4pGWB67xx-MD8 series contains UDP *N*-acetylglucosamine: dolichol phosphate *N*-acetylglucosamine-1-P transferase (GPT) providing tunicamycin resistance (Tunica^r^) (Fig 2A). The availability of the four selection markers in this vector system is useful for transformation experiments and is of particular importance for repetitive transformation in order to introduce new transgenes into previously generated transgenic plants. All marker genes are driven by the nopaline synthase promoter (Pnos) and followed by Tnos. The last two digits represent the type of tag incorporated into these vectors. Seventeen different tags, visible reporters and epitope tags, were equipped in this system for fusion to both ORFs (Fig 2E), namely; synthetic green fluorescent protein (sGFP) [22], the hexahistidine tag (6xHis) [23], FLAG tag (FLAG) [24], triple HA tag (3xHA), four or ten repeats of the Myc tag (4xMyc or 10xMyc) [23], glutathione S-transferase (GST) [25], the T7 epitope tag (T7) [26], β-glucuronidase (GUS) [27], luciferase (LUC) [28], enhanced yellow fluorescent protein (EYFP), enhanced cyan fluorescent protein (ECFP) [29], G3 green fluorescent protein (G3GFP) [30], monomeric red fluorescent protein (mRFP) [31], tag red fluorescent protein (TagRFP) [32], and the N- and C-terminal fragments of enhanced yellow fluorescent protein (nYFP and cYFP) [33]. With these tags, co-purification and co-immuno-precipitation analyses using different epitope tags, comparisons of the expression of two genes and the localization of gene products simultaneously using different visual reporter genes, and protein-protein interaction studies with bimolecular fluorescent complementation using nYFP and cYFP are possible. The two-digit number corresponding to each tag is consistent with that of R4 Gateway Binary Vectors (R4pGWBs) [21, 34–36].

The R4DD vectors with the MD8 sequence were named R4pDD6xx-MD8 (Fig 2B and 2D). The first digit “6” of the three-digit number indicates the destination donor-type vector compatible with R4pGWB6xxx-MD8. The last two digits indicate the types of tags that are consistent with R4DSB vectors.

Most previous vector systems, particularly those for multi-gene expression, were equipped with a limited number of commonly used constitutive promoters such as the cauliflower mosaic virus 35S promoter, *A*. *thaliana UBIQUITIN10* promoter, and *A*. *thaliana ACTIN2* promoter [37–39]. These promoters are often pre-cloned in vectors, and replacing them with other promoters is difficult and requires extra cloning steps. However, the expression of transgenes by various promoters, such as their own promoters, inducible promoters, tissue- or cell-specific promoters, and developmental stage-specific promoters, is required in many plant transgenic experiments. In the R4DS Gateway cloning system, many types of promoter entry clones (*att*L4-promoter-*att*R1) that have accumulated in the plant research community [21, 40–43] can be used for the desired expression of two transgenes. Also, these promoter entry clones are compatible with the large number of ORF entry clones (*att*Ll-ORF-*att*L2) currently available [41, 42, 44, 45]. Various two-gene constructs can be easily generated by LR reactions with the combination of two promoters, two ORFs, and seventeen tags in this system.

The complete nucleotide sequences of R4pGWB6xxx-MD8 and R4pDD6xx-MD8 are available in the GenBank/EML/DDBJ databases under the accession numbers indicated in S1 Table. R4DD and R4DSB vectors developed in this work will be available through RIKEN BioResource Center (http://epd.brc.riken.jp/en/).

### Construction of two-gene fusion driven by stomatal lineage-specific promoters for the visualization of intracellular organelles

**Fig 3:**
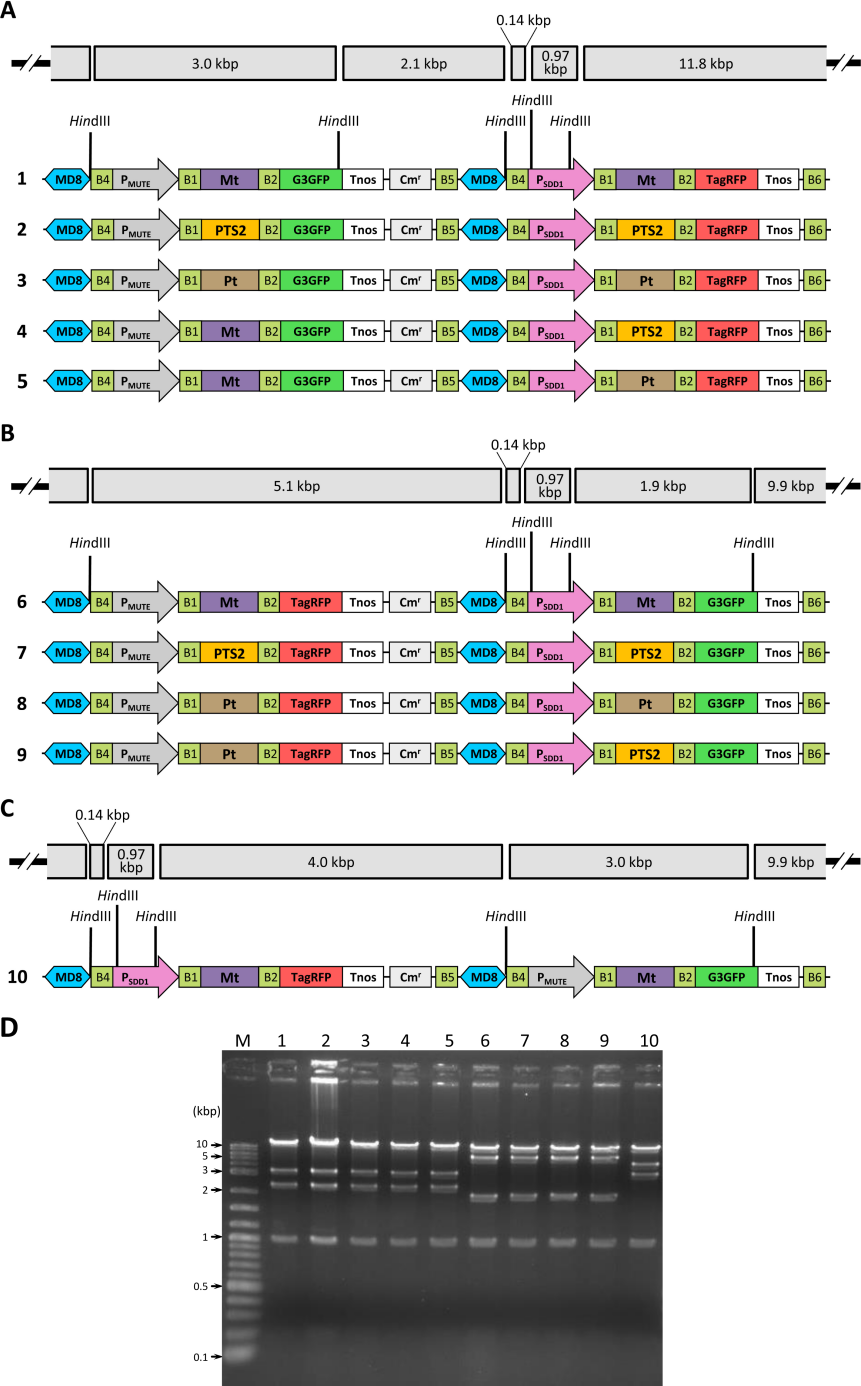
Illustration of ten R4pGWB64xx-MD8 constructs carrying Pro1:ORF1-tag1-Pro2:ORF2-tag2 and confirmation of structures by restriction digestion. (A) Structure of P_MUTE_:ORF1-G3GFP-P_SDD1_:ORF2-TagRFP constructed with R4pGWB6450-MD8 and R4pDD659-MD8 (binary clones 1–5). (B) Structure of P_MUTE_:ORF1-TagRFP-P_SDD1_:ORF2-G3GFP constructed with R4pGWB6459-MD8 and R4pDD650-MD8 (binary clones 6–9). (C) Structure of P_SDD1_:Mt-TagRFP-P_MUTE_:Mt-G3GFP constructed with R4pGWB6459-MD8 and R4pDD650-MD8 (binary clone 10). The positions of *Hin*dIII sites are indicated and the sizes of restriction fragments are shown in kilobase pairs (kbp). (D) Binary clones 1–10 were digested by *Hin*dIII and electrophoresed on 1.5% agarose gel. Lanes 1–10 show binary clones 1–10. Lane M shows the DNA ladder marker. The positions of 10, 5, 3, 2, 1, 0.5, and 0.1 kbp are indicated. P_MUTE_, *MUTE* promoter; P_SDD1_, *SDD1* promoter; Mt, mitochondria-targeting signal; PTS2, peroxisome-targeting signal type 2; Pt, plastid-targeting signal.

In order to test the performance of the R4DS Gateway cloning system, we employed a combination of two stomatal lineage-specific promoters, *MUTE* promoter (P_MUTE_) [46, 47] and *STOMATAL DENSITY AND DISTRIBUTION1* (*SDD1*) promoter (P_SDD1_) [48, 49], and three organelle-targeting signals, a mitochondria-targeting signal of the *A*. *thaliana* F_1_-ATPase γ subunit (Mt) [50], peroxisome-targeting signal type 2 of pumpkin citrate synthase (PTS2) [51], and a plastid-targeting signal of the *A*. *thaliana* RuBisCO small subunit (Pt) [52]. We selected R4pDD650-MD8 (G3GFP) and R4pDD659-MD8 (TagRFP) as R4DD vectors, and R4pGWB6450-MD8 (G3GFP) and R4pGWB6459-MD8 (TagRFP) as R4DSB vectors. By using successive first and second LR reactions, we made 10 constructs carrying two sets of organelle-targeting signal-fluorescent protein genes driven by stomatal lineage-specific promoters (Fig 3, S2 Table). In order to increase recombination efficiency, the R4DD and R4DSB vectors were linearized prior to the LR reaction. We summarized the recommended restriction enzymes for the linearization of R4pDD6xx-MD8 and R4pGWB6xxx-MD8 in S1 Table. In order to confirm the structure of the ten binary constructs, we analyzed them by restriction digestion. As shown in gel electrophoresis (Fig 3D), DNA fragments generated by *Hin*dIII digestion from all tested clones migrated to the positions of the expected sizes, as shown in Fig 3A, 3B, and 3C and S2 Table, indicating the successful preparation of two-gene constructs. All these ten two-gene binary clones were constructed with 80–100% success rate. Out of five randomly chosen colonies, four to five were constructed successfully with the two-gene expression cassettes assembled in the desired combination. These clones were introduced into *A*. *tumefaciens*, and then used for the transformation of *A*. *thaliana*. We obtained transgenic lines for all constructs showing segregation of 3:1 on kanamycin selection plates in the T2 generation. These lines indicating the integration of transgenes at a single locus were used in subsequent experiments.

### Promoter-directed expression and organelle targeting of fluorescent proteins in transgenic *A*. *thaliana*

**Fig 4:**
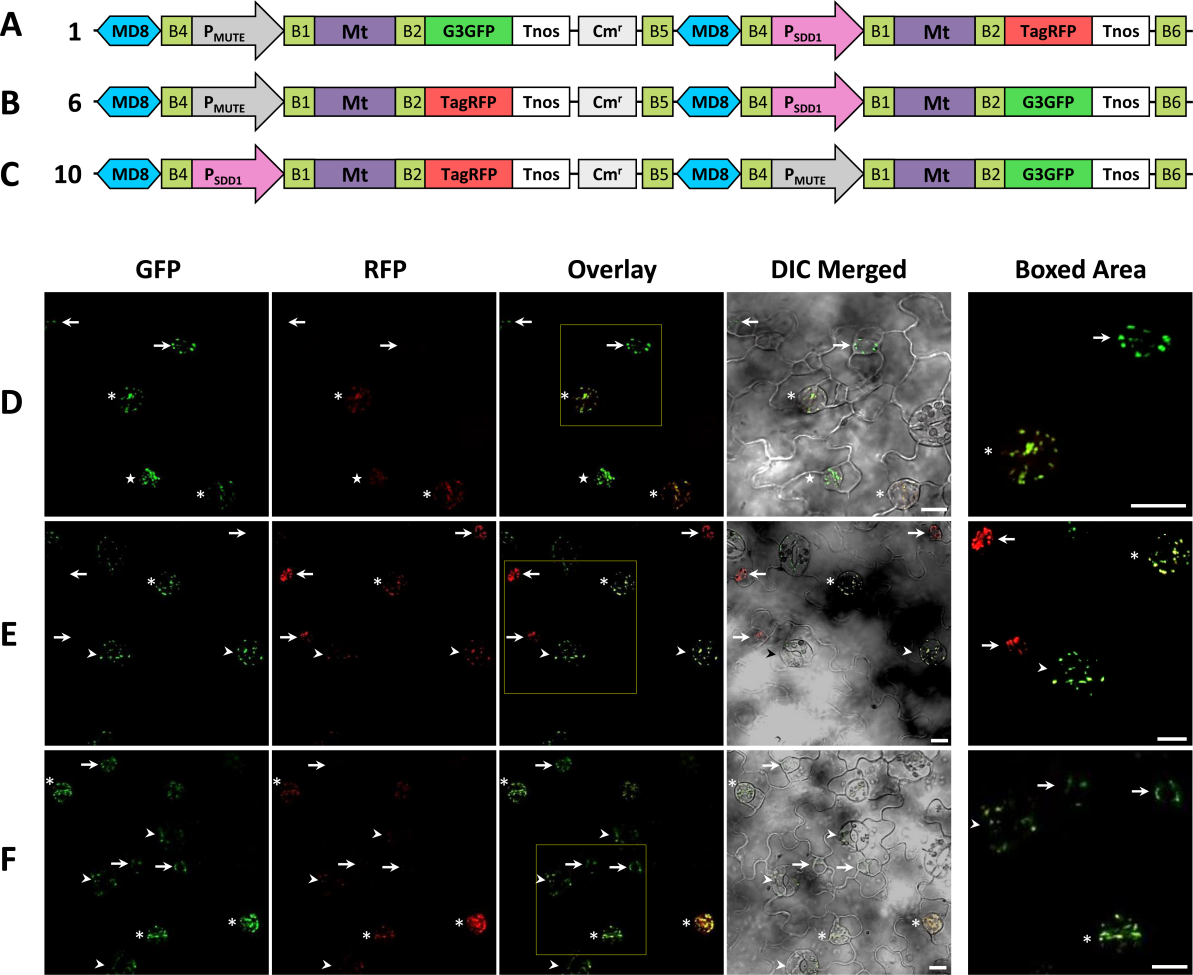
Expression and intracellular localization of G3GFP and TagRFP fused with the mitochondria-targeting signal in transformed *A*. *thaliana* with different arrangements of promoters and cloning positions. (A-C) Structural diagram of the binary constructs 1, 6, and 10 used in expression experiments. (D-F) Fluorescent images of the leaf epidermis of *A*. *thaliana* transformed with construct 1 (D), 6 (E), or 10 (F). Different developmental stages in stomatal lineages are indicated by arrows (meristemoids), stars (GMCs), asterisks (immature GCs), or arrowheads (mature GCs). In meristemoids, only the expression of the fluorescent protein directed by P_MUTE_ was observed, while both signals were detected in later stages. GFP, signal of G3GFP; RFP, signal of TagRFP; Overlay, overlay of GFP and RFP; DIC Merged, differential interference contrast (DIC) merged with GFP and RFP; Boxed Area, enlargement of the boxed area in the overlay. Scale bars = 10 μm.

**Fig 5:**
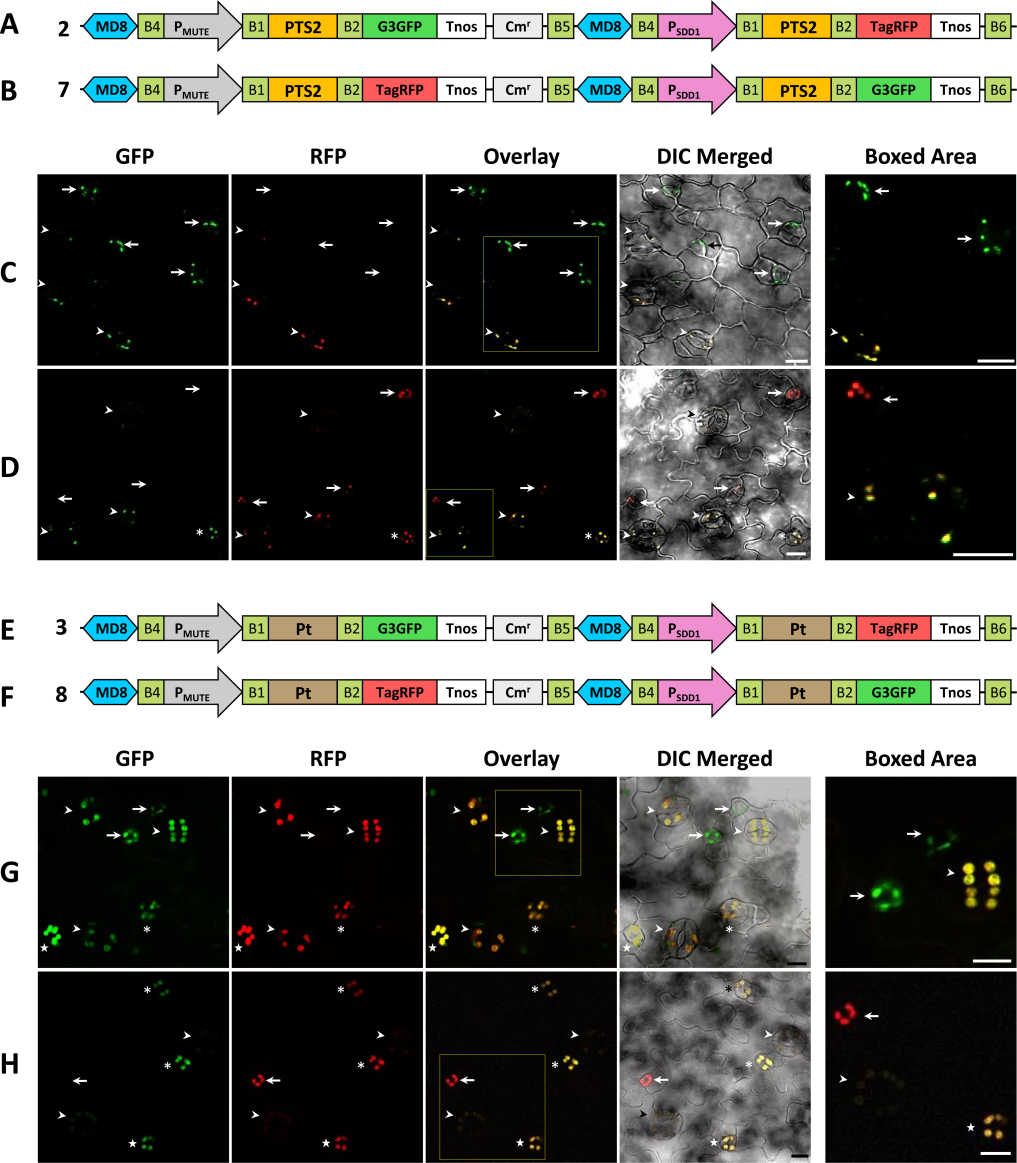
Expression and intracellular localization of G3GFP and TagRFP fused with peroxisome- or plastid-targeting signals in transformed *A*. *thaliana* with different combinations of promoters. (A, B) Structural diagram of the binary constructs with peroxisome-targeting signals (constructs 2 and 7). (C, D) Fluorescent images of the leaf epidermis of *A*. *thaliana* transformed with construct 2 (C) or 7 (D). (E, F) Structural diagram of the binary constructs with plastid-targeting signals (constructs 3 and 8). (G, H) Fluorescent images of the leaf epidermis of *A*. *thaliana* transformed with construct 3 (G) or 8 (H). Different developmental stages in stomatal lineages are indicated by arrows (meristemoids), stars (GMCs), asterisks (immature GCs), or arrowheads (mature GCs). In meristemoids, only the expression of the fluorescent protein directed by P_MUTE_ was observed, while both signals were detected in later stages. GFP, signal of G3GFP; RFP, signal of TagRFP; Overlay, overlay of GFP and RFP; DIC Merged, differential interference contrast (DIC) merged with GFP and RFP; Boxed Area, enlargement of the boxed area in the overlay. Scale bars = 10 μm.

Confocal laser scanning microscopy was used to detect the fluorescent signals of G3GFP and TagRFP in transgenic *A*. *thaliana* plants. We analyzed the co-expression rate of two fluorescent proteins in 10 lines for each construct (constructs 1 to 10 in Fig 3) and detected the expression of G3GFP and TagRFP in all lines examined. Binary clones constructed using the R4DS Gateway cloning system showed markedly higher co-expression efficiencies under the experimental conditions described herein than previously reported two-gene expression vectors that showed 10 to 88% of the co-expression efficiency of the two transgenes in transformed *A*. *thaliana* [37–39]. All the observed lines showed the expression of both fluorescent proteins, indicating 100% co-expression efficiency. We then analyzed the precise expression timing and subcellular localization of G3GFP and TagRFP for each construct carrying a combination of two stomatal lineage-specific promoters (*MUTE* and *SDD1* promoters) and three organelle-targeting signals (mitochondria-, peroxisome-, and plastid-targeting signals). In *A*. *thaliana*, the development of stomata goes through a specialized cell lineage, stomatal lineage, which consists of the following five stages; meristemoid mother cells, meristemoids, guard mother cells (GMCs), immature guard cells (immature GCs), and mature GCs [53, 54]. MUTE is a basic helix–loop–helix protein that plays a role in the termination of stem cell behavior by triggering differentiation from meristemoids to GMCs. P_MUTE_ initiates the expression of downstream genes at the meristemoid stage [46]. SDD1 is a subtilisin-like serine protease that negatively regulates stomatal density [48]. P_SDD1_ is activated slightly later than P_MUTE_ around the GMC stage [49]. We demonstrated the difference of P_MUTE_ and P_SDD1_ activities by showing the fluorescence of mitochondria-targeted G3GFP and TagRFP in stomatal lineage cells in the leaf epidermis. Fig 4A shows the construct, P_MUTE_:Mt-G3GFP-P_SDD1_:Mt-TagRFP (construct 1 in Fig 3), carrying a mitochondria-targeting signal fused with G3GFP (Mt-G3GFP) under the control of P_MUTE_ at the first cloning site (the cloning site upstream of Cm^r^) and mitochondria-targeting signal fused with TagRFP (Mt-TagRFP) under the control of P_SDD1_ at the second cloning site (the cloning site downstream of Cm^r^). The second construct used for comparison was P_MUTE_:Mt-TagRFP-P_SDD1_:Mt-G3GFP (construct 6), in which Mt-G3GFP and Mt-TagRFP of construct 1 were replaced with each other (Fig 4B). At the meristemoid stage, only G3GFP was detected in the mitochondria of plants transformed with construct 1, while only TagRFP was detected in the mitochondria of plants transformed with construct 6. At the GMC and immature GC stages and weakly at the mature GC stage, G3GFP and TagRFP were both observed in the mitochondria of plants transformed with both constructs (Fig 4D and 4E). These results coincide with the expression patterns of the *MUTE* and *SDD1* genes. Similar results were obtained in experiments in which the mitochondria-targeting signal was replaced with the peroxisome-targeting signal [P_MUTE_:PTS2-G3GFP-P_SDD1_:PTS2-TagRFP (construct 2, Fig 5A and 5C) and P_MUTE_:PTS2-TagRFP-P_SDD1_:PTS2-G3GFP (construct 7, Fig 5B and 5D)] or with the plastid-targeting signal [P_MUTE_:Pt-G3GFP-P_SDD1_:Pt-TagRFP (construct 3, Fig 5E and 5G) and P_MUTE_:Pt-TagRFP-P_SDD1_:Pt-G3GFP (construct 8, Fig 5F and 5H)]. In order to confirm that differences in expression timing are not dependent on the position of the two cloning sites in the vectors, the location of expression units were exchanged in the constructs [P_MUTE_:Mt-G3GFP-P_SDD1_:Mt-TagRFP (construct 1, Fig 4A) to P_SDD1_:Mt-TagRFP-P_MUTE_:Mt-G3GFP (construct 10, Fig 4C)]. The same expression patterns were observed in both constructs (Fig 4D and 4F), indicating that the cloning sites of R4DSB vectors are equivalent.

**Fig 6:**
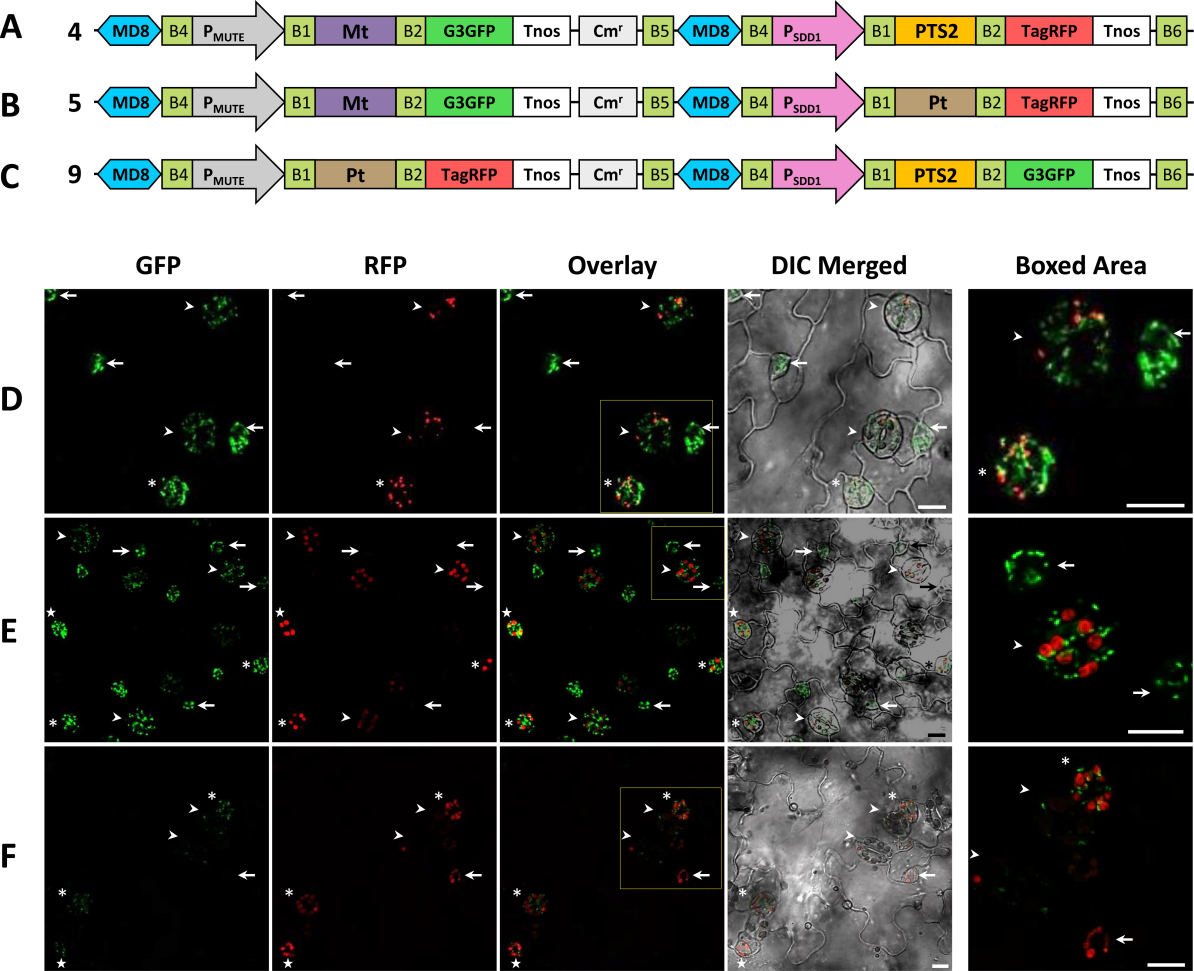
Expression and intracellular localization of G3GFP and TagRFP fused with different combinations of mitochondria-, peroxisome-, and plastid-targeting signals in transformed *A*. *thaliana* with different promoters. (A-C) Structural diagram of the binary constructs 4, 5, and 9 with different targeting signals. (D-F) Fluorescent images of the leaf epidermis of *A*. *thaliana* transformed with construct 4 (D), 5 (E), or 9 (F). Different developmental stages in stomatal lineages are indicated by arrows (meristemoids), stars (GMCs), asterisks (immature GCs), or arrowheads (mature GCs). In meristemoids, only the expression of the fluorescent protein directed by P_MUTE_ was observed, while both signals were detected in later stages. GFP, signal of G3GFP; RFP, signal of TagRFP; Overlay, overlay of GFP and RFP; DIC Merged, differential interference contrast (DIC) merged with GFP and RFP; Boxed Area, enlargement of the boxed area in the overlay. Scale bars = 10 μm.

We also examined the constructs carrying G3GFP and TagRFP fused with different organelle-targeting signals and driven by different promoters. In plants transformed with construct 4 (P_MUTE_:Mt-G3GFP-P_SDD1_:PTS2-TagRFP, Fig 6A), only the mitochondria-targeted G3GFP signal was detected in meristemoids. In GMCs, immature GCs, and weakly in mature GCs, the G3GFP signal and TagRFP signal were both detected, but localized in different subcellular compartments (Fig 6D). P_MUTE_:Mt-G3GFP-P_SDD1_:Pt-TagRFP (construct 5, Fig 6B) and P_MUTE_:Pt-TagRFP-P_SDD1_:PTS2-G3GFP (construct 9, Fig 6C) also showed consistent fluorescence images corresponding to their targeting signals and promoters (Fig 6E and 6F). These results indicate that the two genes cloned by the R4DS Gateway cloning system are independently regulated.

In the present study, we developed the R4DS Gateway cloning system with the following desirable features; (1) Compatible with universal-type entry clones (*att*L4-promoter-*att*R1 and *att*L1-ORF-*att*L2); therefore, the resources of promoter and ORF entry clones in the research community can be directly used in any combination for cloning. (2) Two-gene cloning can be accomplished easily using two rounds of Gateway LR reactions. (3) The 17 different tags including visible proteins and epitope tags can be fused at the C-terminal of each ORF in any combination. (4) Four kinds of resistance markers conferring Km^r^, Hyg^r^, BASTA^r^, and Tunica^r^ are available, and, thus, are useful for the re-transformation of plants already carrying a selection marker(s). We made 10 different two-gene constructs using R4pGWB6xxx-MD8 and found efficient and accurate cloning performance. Expression experiments with P_MUTE_ and P_SDD1_ confirmed the promoter-specific independent expression of two ORFs, regardless of the combination of the promoters used or position of cloning sites, in *A*. *thaliana*. The R4DS Gateway cloning system is a powerful tool for plant transformation and will contribute to a better understanding of molecular gene functions and protein intracellular dynamics, e.g., comparing two transgene expression patterns, elucidating subcellular co-localization, analyzing protein complex formation, and detecting protein-protein interactions.

## Materials and methods

### DNA manipulation and plasmid construction

All plasmids described in this study were handled according to standard DNA manipulation methods [55]. In PCR, KOD DNA polymerase (TOYOBO, Osaka, Japan) was used to amplify products with blunt ends. The nucleotide sequences of cloned PCR fragments, synthetic oligo DNAs, and their ligated junctions in all intermediate and final vectors were confirmed by sequencing. All oligonucleotides used in this study as a linker, adaptors and primers are listed in S3 Table.

We used pGWB400, 500 [56], 600 [35], and 700 [36] binary vectors, which are based on the pPZP vector [57], as a backbone to create R4pGWB6xxx-MD8. In brief, *att*R4-*ccd*B-*att*R2 and *att*R5-*ccd*B-*att*R6 sequences were generated on binary vectors as the first and second acceptor sites for Gateway cloning, respectively. The R4pDD6xx-MD8 series was constructed on the pUC119 vector by generating the *att*R4-Cm^r^-*ccd*B-*att*R2 sequence nested between *att*L5 and *att*L6. MD8, a MAR of *A*. *thaliana* [14], was placed upstream of each *att*R4 site (cloning site for the promoter) in order to provide more stability for transgene expression. Plasmid construction and cloning strategies are described in detail in S1 Text and S1 Fig.

### Construction of entry clones and final expression vectors

P_MUTE_ was amplified from *A*. *thaliana* (ecotype Col-0) wild type genomic DNA using P_MUTE_-*att*B4 and P_MUTE_-*att*B1 primers (S3 Table) to add the *att*B4 and *att*B1 sequences to its 5' and 3' ends, respectively. The resulting fragment was subjected to a BP reaction with pDONRP4-P1R (Invitrogen) to construct the *att*L4-P_MUTE_-*att*R1 entry clone. PCR and BP reactions were performed according to the manufacturer’s instructions (Invitrogen). The *att*L4-P_SDD1_-*att*R1 entry clone was prepared as described previously [21]. In the construction of ORF entry clones, organelle-targeting sequences were PCR amplified from cDNA prepared from Col-0 wild-type seedlings that germinated for 5 days under dark conditions and 2 days under light conditions. The DNA fragment corresponding to the N-terminal amino acids (1–42) of the *A*. *thaliana* F_1_-ATPase γ subunit (mitochondria-targeting signal) was amplified using F_1_ATPg-*att*B1 and F_1_ATPg-*att*B2 primers (S3 Table). The amplified DNA fragment was further used in a second adaptor PCR with *att*B1 adaptor and *att*B2 adaptor primers (S3 Table) to add the *att*B1 and *att*B2 sequences to its 5' and 3' ends, respectively. The resulting fragment was subjected to the BP reaction with pDONR221 (Invitrogen) in order to construct the pF1gLS221 entry clone. Similarly, the DNA fragment corresponding to the N-terminal amino acids (1–55) of the *A*. *thaliana* RuBisCO small subunit (plastid-targeting signal) was amplified with the RBCS1A-*att*B1 and RBCS1A-*att*B2 primers and then with *att*B1 adaptor and *att*B2 adaptor primers (S3 Table) and used for the construction of pRbcSTP221. F_1_ATPg-*att*B2 and RBCS1A-*att*B2 primers were designed to insert one nucleotide between the last codon of ORF and the *att*B2 (*att*L2 in the entry clone) according to the manufacturer’s instructions (Invitrogen) for in-frame fusion with fluorescent protein encoded in destination vector.

In the construction of two sets of the promoter:ORF-fluorescent protein genes on R4DSB vectors, the following two rounds of LR reactions were performed. *att*L4-P_MUTE_-*att*R1 and *att*L4-P_SDD1_-*att*R1 were used as the promoter entry clones. pF1gLS221, pRbcSTP221, and pPTS2-221 [58] were used as the ORF entry clones. In the first LR reaction, a promoter entry clone, ORF entry clone, and linearized R4pDD650-MD8 (or R4pDD659-MD8) by *Sal*I digestion were subjected to a tripartite LR reaction [21] in order to make the promoter:ORF-tag fusion gene on R4DD vectors (R4pDD6xx-MD8-Pro2:ORF2 in Fig 1A). In the second LR reaction, a quadripartite LR reaction was performed using the following two-step procedure. R4pDD6xx-MD8-Pro2:ORF2 and linearized R4pGWB6450-MD8 (or R4pGWB6459-MD8) by *Sal*I digestion were subjected to the LR reaction. At the same time a promoter entry clone and ORF entry clone were subjected to the LR reaction. After an incubation for 5 hours, these two solutions were mixed and incubated further overnight with a boost of LR clonase to construct a binary clone carrying two sets of the promoter:ORF-tag fusion (R4pGWB6xxx-MD8-Pro1:ORF1-Pro2:ORF2 in Fig 1B). Ten different combinations of Pro1:ORF1-tag1-Pro2:ORF2-tag2 were constructed as final binary clones (Fig 3 and S2 Table). The full procedure for constructing and confirming the two-gene constructs took less than one week (excluding the time required for preparing entry clones). For each of the first and second LR reaction, one day is required for vector construction and two days for vector confirmation.

### Vector manipulation in *Escherichia coli* and *A. tumefaciens*

*E*. *coli* strains DH5α or One Shot^®^ *ccd*B survival™ 2T1^R^ (Invitrogen, Life Technologies) were used to construct plasmids. The transformation of *A*. *tumefaciens* strain C58C1 (pMP90) was performed by electroporation. *E*. *coli* with the recombinant plasmids was grown at 37°C for approximately 12 to 16 h in Luria broth (LB) media supplemented with appropriate antibiotics. *A*. *tumefaciens* was grown at 28° C for 48–72 h in LB media supplemented with the appropriate antibiotics.

### Transformation and growth of *A*. *thaliana*

*A*. *tumefaciens* strains with the recombinant R4DSB vectors were used to transform *A*. *thaliana* ecotype Col-0 using the floral dip transformation method [59], and treated plants were allowed to set seeds at 22°C under a long-day photoperiod (16/8-hr light/dark cycle). The seeds were vernalized at 4°C for 3 days and selected on Murashige and Skoog (MS) agar medium containing kanamycin (30 mg /L) and cefotax (100 mg /L) under 24-hr continuous light conditions for two weeks. Fourteen-day-old seedlings of the selected lines (T1) were transplanted to soil pots and allowed to grow at 22°C under a long-day photoperiod. T2 seeds were collected and the segregation of transgenes was analyzed by selection on MS medium containing kanamycin and cefotax.

### Confocal microscopy

The abaxial surfaces of the fully expanded leaves of two-week-old T1 or T2 seedlings were viewed with a TCS SP5 confocal laser-scanning microscope (Leica Microsystems, Wetzlar, Germany) using an HCX PL APO CS 20.0 x 0.7 IMM UV water immersion objective lens. G3GFP was excited with the argon laser line (488 nm) and TagRFP was excited with the helium-neon laser line (543 nm). The fluorescence of G3GFP and TagRFP was detected using the emission filters, BP500-530 nm and BP555-615 nm, respectively. Images were acquired sequentially line-by-line with a resolution of 512×512 pixels and 400-Hz scanning speed. Image analyses were processed in Adobe Photoshop 8.0 (Adobe Systems Incorporated, CA, U.S.A.) and converted to the TIFF format.

## Supporting information

S1 Fig Representative scheme for the construction of R4pGWB6xxx-MD8 and R4pDD6xx-MD8. Numbers in parentheses indicate the position of restriction sites in *BAGEL7* (1899 bp). Final constructs are indicated by red letters. Amp^r^, ampicillin resistance; Cm^r^, chloramphenicol resistance; Cm, a part of the Cm^r^ marker; Km^r^, kanamycin resistance; *ccd*B, negative selection marker; Tnos, nopaline synthase terminator; P35S, cauliflower mosaic virus 35S promoter; L1, *att*L1; L2, *att*L2; L3, *att*L3; L4, *att*L4; L5, *att*L5; L6, *att*L6; R1, *att*R1; R2, *att*R2; R3, *att*R3; R4, *att*R4; R5, *att*R5; R6, *att*R6; Ap, *Apa*I; As, *Asc*I; Bs, *Bsp*EI; E, *Eco*RI; H, *Hin*dIII; Hp, *Hpa*I; M, *Msc*I; N, *Not*I; Nc, *Nco*I; Nr, *Nru*I; Sc, *Sac*I; Sm, *Sma*I; Sw, *Swa*I; Xb, *Xba*I; Xh, *Xho*I. References are listed in S1 Text.
(PDF)

S1 Table Backbone, bacterial selection, plant selection, tag, restriction enzyme for linearization, and accession numbers of R4pDD6xx-MD8 and R4pGWB6xxx-MD8. Amp^r^, ampicillin resistance; Cm^r^, chloramphenicol resistance; Spc^r^, spectinomycin resistance; the NPTII gene for kanamycin resistance (Km^r^), the HPT gene for hygromycin B resistance (Hyg^r^), the bar gene for BASTA resistance (BASTA^r^), and the GPT gene for tunicamycin resistance (Tunica^r^).
(DOCX)

S2 Table Binary clones constructed in this study. Ten binary clones containing different combinations of Pro1:ORF1-tag1-Pro2:ORF2-tag2 were constructed. The total vector size and size of *Hin*dIII fragments are indicated in base pairs (bp). P_MUTE_, *MUTE* promoter; P_SDD1_, *SDD1* promoter; Mt, mitochondria-targeting signal; PTS2, peroxisome-targeting signal type 2; Pt, plastid-targeting signal.
(DOCX)

S3 Table Oligonucleotides used in this study. Restriction sites are underlined.
(DOCX)

S1 Text A detailed description of plasmid construction. (DOCX)
