# Supplementary material for: Development of an R4 dual-site (R4DS) gateway cloning system enabling the efficient simultaneous cloning of two desired sets of promoters and open reading frames in a binary vector for plant research
Source: PLoS One. 2017 May 16;12(5):e0177889. doi: 10.1371/journal.pone.0177889 (PMC5433782; doi:10.1371/journal.pone.0177889)
Supplement: S1 Fig — Numbers in parentheses indicate the position of restriction sites in BAGEL7 (1899 bp). Final constructs are indicated by red letters. Ampr, ampicillin resistance; Cmr, chloramphenicol resistance; Cm, a part of the Cmr marker; Kmr, kanamycin resistance; ccdB, negative selection marker; Tnos, nopaline synthase terminator; P35S, cauliflower mosaic virus 35S promoter; L1, attL1; L2, attL2; L3, attL3; L4, attL4; L5, attL5; L6, attL6; R1, attR1; R2, attR2; R3, attR3; R4, attR4; R5, attR5; R6, attR6; Ap, ApaI; As, AscI; Bs, BspEI; E, EcoRI; H, HindIII; Hp, HpaI; M, MscI; N, NotI; Nc, NcoI; Nr, NruI; Sc, SacI; Sm, SmaI; Sw, SwaI; Xb, XbaI; Xh, XhoI. References are listed in S1 Text. (PDF) [file pone.0177889.s001.pdf]

Construction of R4pGWB6x01-MD8 (1)

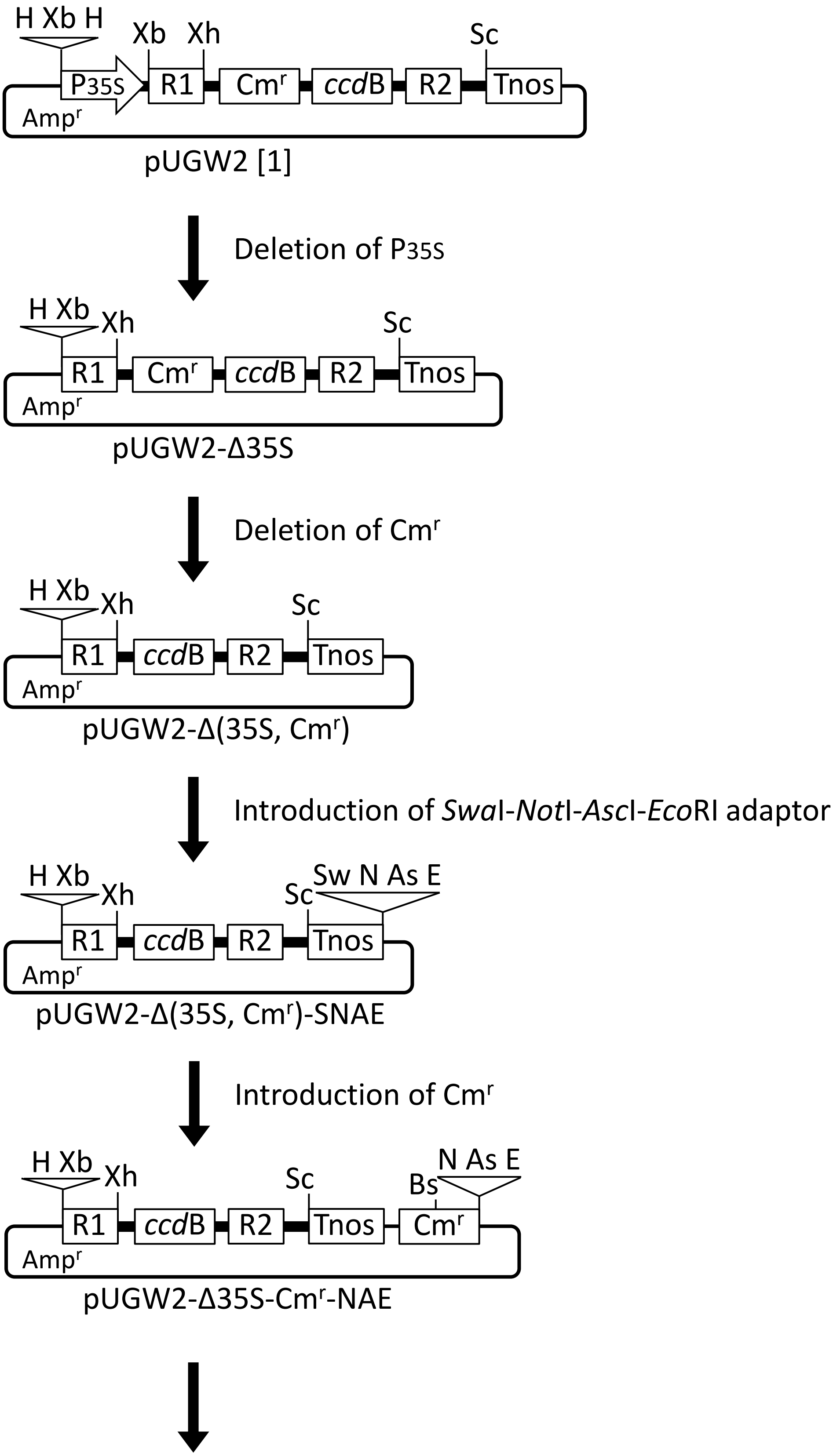

Next page

Construction of R4pGWB6x01-MD8 (2)

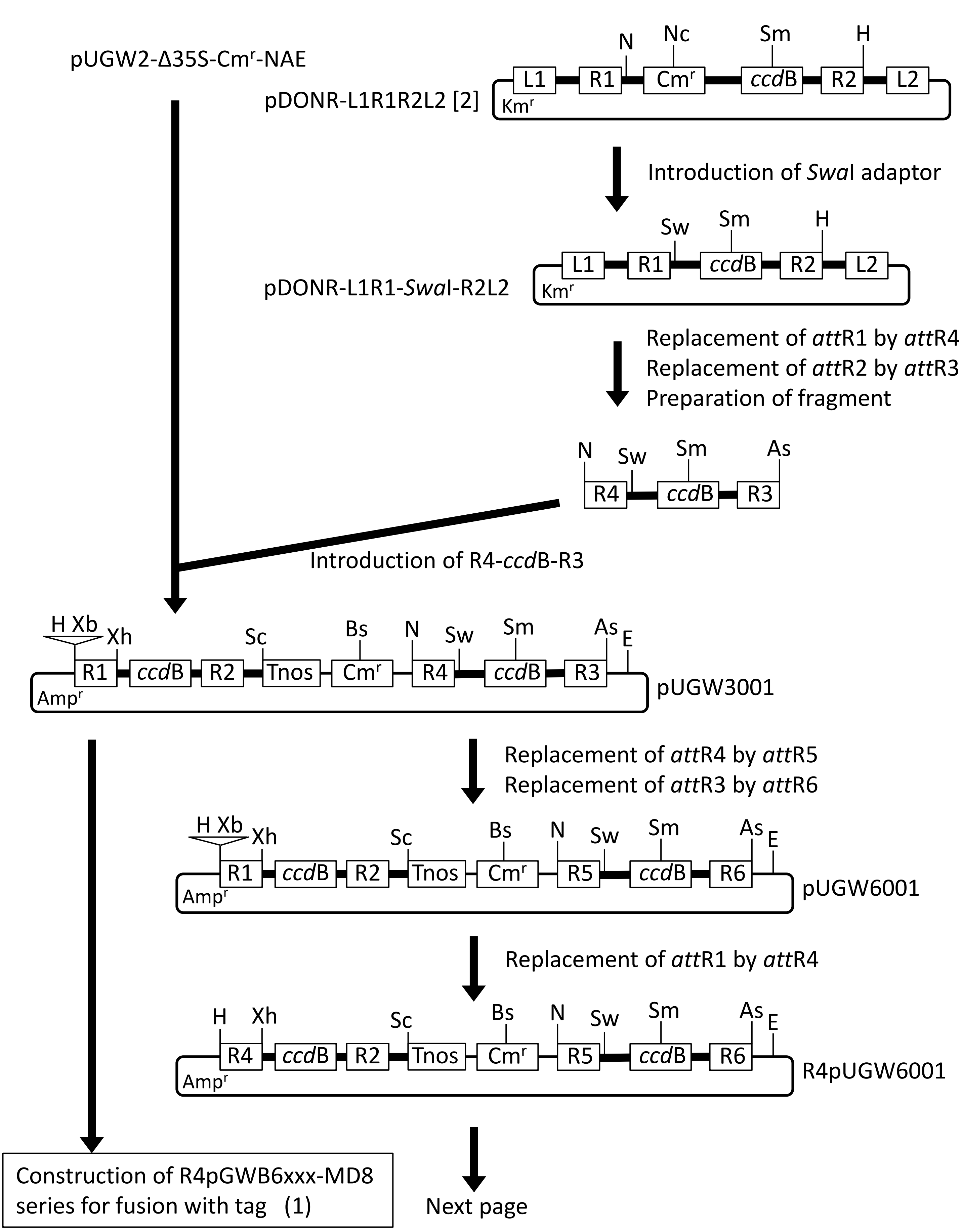

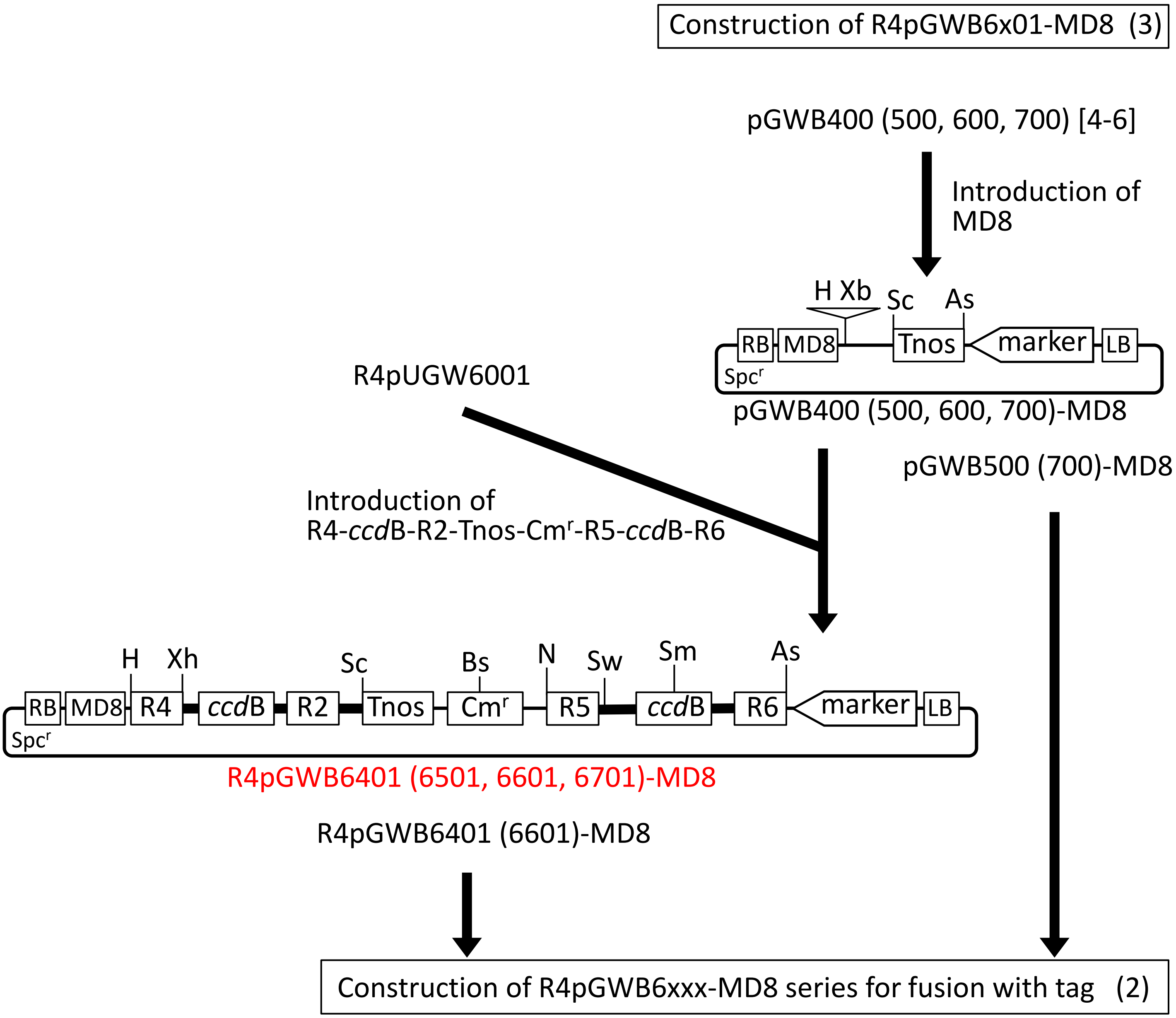
Construction of R4pGWB6x01-MD8 (3)
pGWB400 (500, 600, 700) [4-6]
Introduction of MD8
H Xb
Sc
As
RB
MD8
Tnos
marker
LB
Spcr
pGWB400 (500, 600, 700)-MD8
R4pUGW6001
pGWB500 (700)-MD8
Introduction of R4-ccdB-R2-Tnos-Cmr-R5-ccdB-R6
H
Xh
Sc
Bs
N
Sw
Sm
As
RB
MD8
R4
ccdB
R2
Tnos
Cmr
R5
ccdB
R6
marker
LB
Spcr
R4pGWB6401 (6501, 6601, 6701)-MD8
R4pGWB6401 (6601)-MD8
Construction of R4pGWB6xxx-MD8 series for fusion with tag (2)

Construction of R4pGWB6xxx-MD8 series for fusion with tag (1)

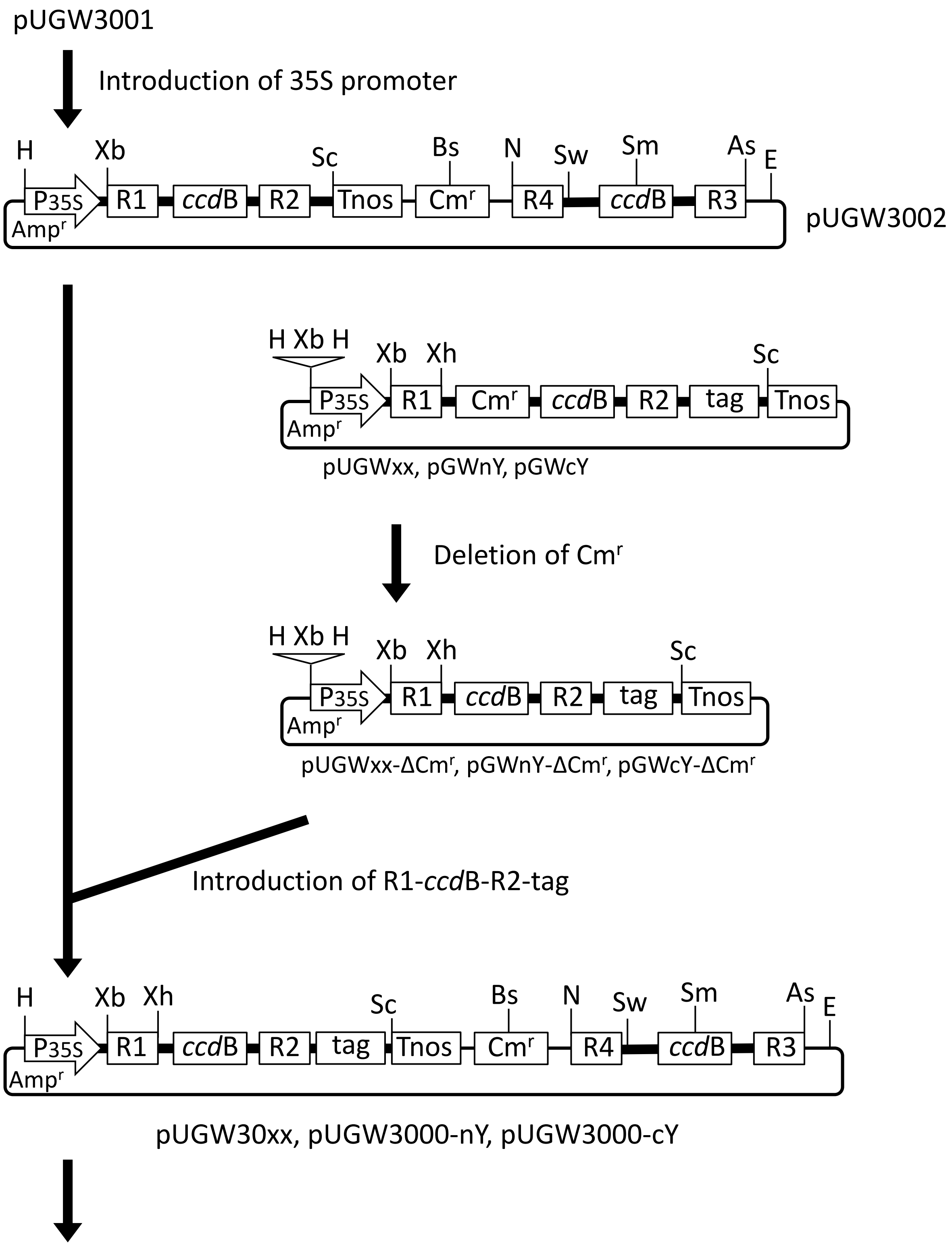

Next page

Construction of R4pGWB6xxx-MD8 series for fusion with tag (2)

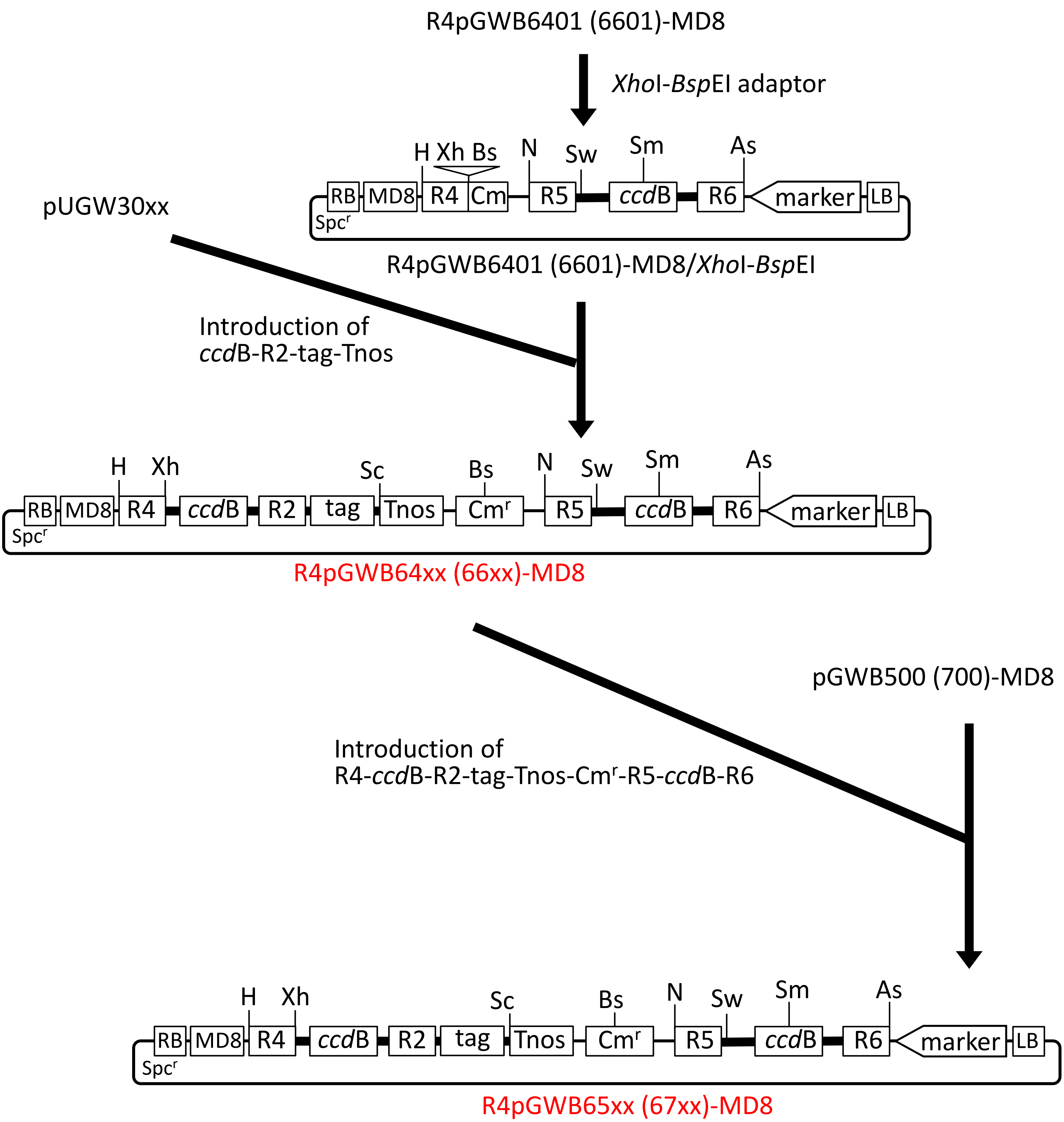

Construction of R4pDD6xx-MD8 (1)

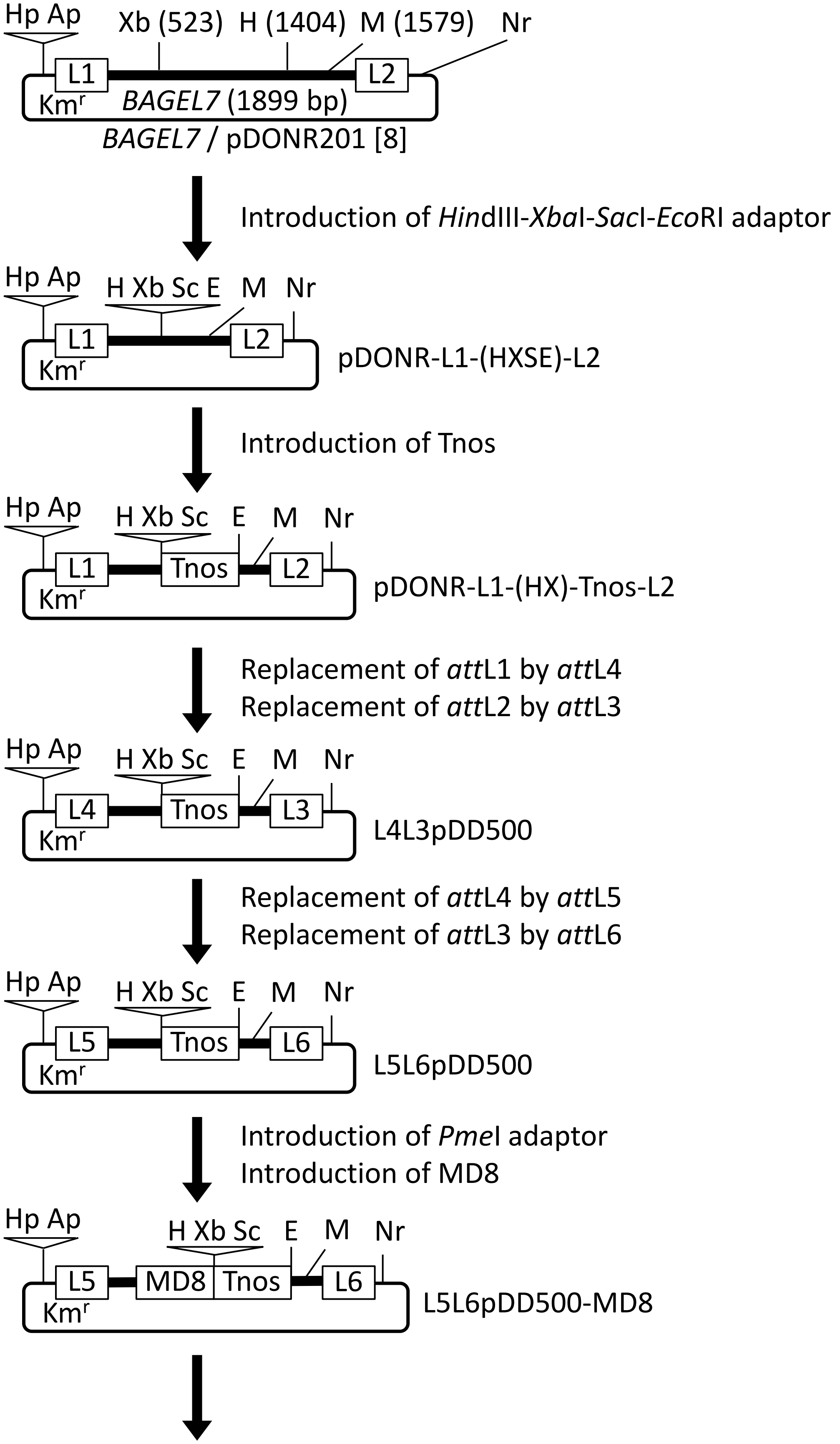

Next page

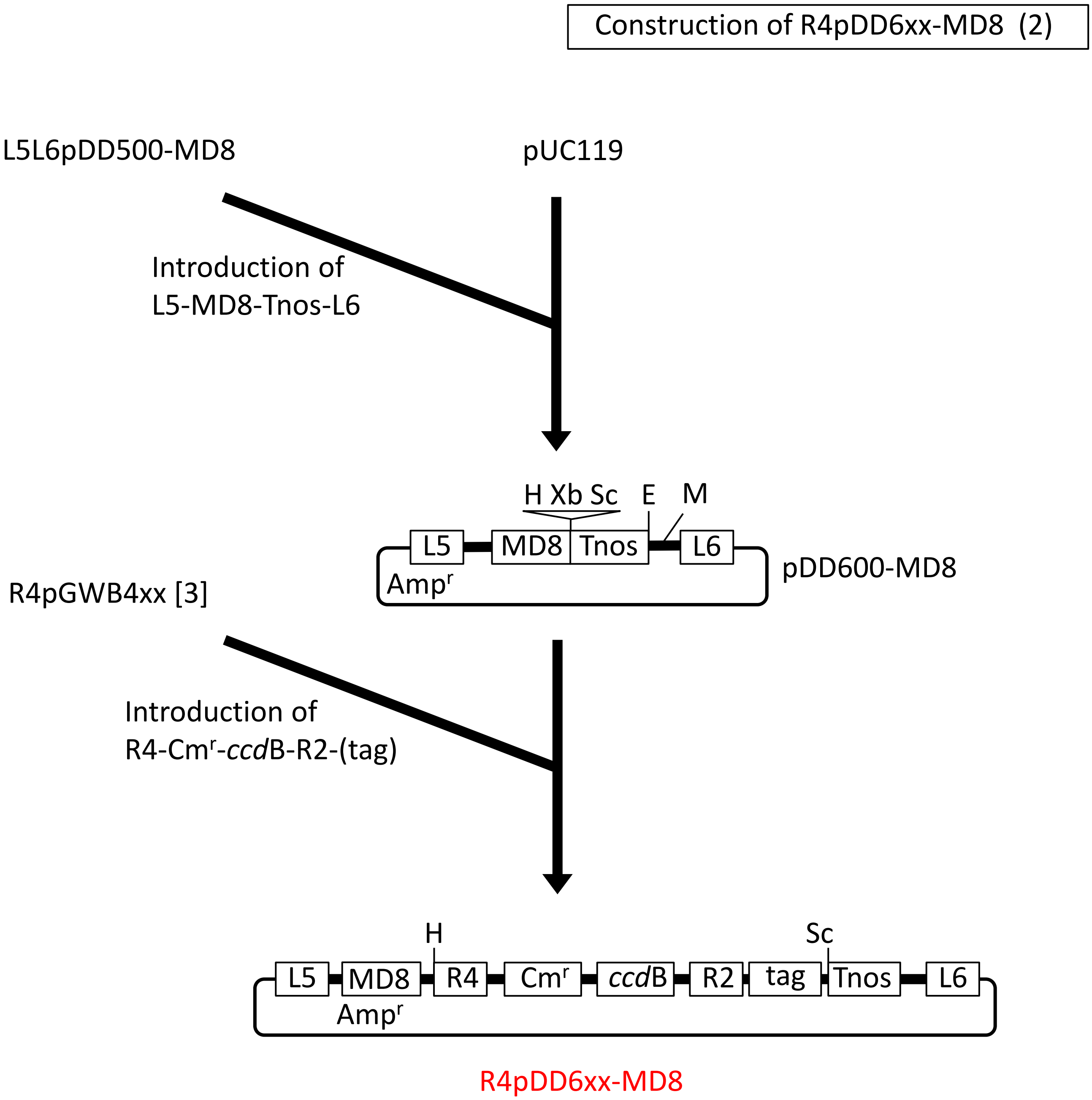

S1 Fig. Representative scheme for the construction of R4pGWB6xxx-MD8 and R4pDD6xx-MD8. Numbers in parentheses indicate the position of restriction sites in *BAGEL7* (1899 bp). Final constructs are indicated by red letters. Amp^r, ampicillin resistance; Cm^r, chloramphenicol resistance; Cm, a part of the Cm^r marker; Km^r, kanamycin resistance; *ccd*B, negative selection marker; Tnos, nopaline synthase terminator; P35S, cauliflower mosaic virus 35S promoter; L1, *att*L1; L2, *att*L2; L3, *att*L3; L4, *att*L4; L5, *att*L5; L6, *att*L6; R1, *att*R1; R2, *att*R2; R3, *att*R3; R4, *att*R4; R5, *att*R5; R6, *att*R6; Ap, *Apa*I; As, *Asc*I; Bs, *Bsp*EI; E, *Eco*RI; H, *Hin*dIII; Hp, *Hpa*I; M, *Msc*I; N, *Not*I; Nc, *Nco*I; Nr, *Nru*I; Sc, *Sac*I; Sm, *Sma*I; Sw, *Swa*I; Xb, *Xba*I; Xh, *Xho*I. References are listed in S1 Text.
