# Supplementary material for: Development of an R4 dual-site (R4DS) gateway cloning system enabling the efficient simultaneous cloning of two desired sets of promoters and open reading frames in a binary vector for plant research
Source: PLoS One. 2017 May 16;12(5):e0177889. doi: 10.1371/journal.pone.0177889 (PMC5433782; doi:10.1371/journal.pone.0177889)
Supplement: S1 Table — Ampr, ampicillin resistance; Cmr, chloramphenicol resistance; Spcr, spectinomycin resistance; the NPTII gene for kanamycin resistance (Kmr), the HPT gene for hygromycin B resistance (Hygr), the bar gene for BASTA resistance (BASTAr), and the GPT gene for tunicamycin resistance (Tunicar). (DOCX) [file pone.0177889.s002.docx]

| **S1 Table. Backbone, bacterial selection, plant selection, tag, restriction enzyme for linearization, and accession numbers of R4pDD6xx-MD8 and R4pGWB6xxx-MD8.** Amp^r^, ampicillin resistance; Cm^r^, chloramphenicol resistance; Spc^r^, spectinomycin resistance; the NPTII gene for kanamycin resistance (Km^r^), the HPT gene for hygromycin B resistance (Hyg^r^), the bar gene for BASTA resistance (BASTA^r^), and the GPT gene for tunicamycin resistance (Tunica^r^). | | | | | | | |
| --- | --- | --- | --- | --- | --- | --- | --- |
| **Plasmid name** | **Backbone** | **Bacterial selection** | **Plant selection** | **Tag** | **Restriction enzyme for linearization** | **Accession number** |  |
| R4pDD601-MD8 | pUC119 | Amp^r^, Cm^r^ | None | None | *Sal*I | LC171733 |  |
| R4pDD604-MD8 | pUC119 | Amp^r^, Cm^r^ | None | sGFP | *Sal*I | LC171734 |  |
| R4pDD607-MD8 | pUC119 | Amp^r^, Cm^r^ | None | 6xHis | *Sal*I | LC171735 |  |
| R4pDD610-MD8 | pUC119 | Amp^r^, Cm^r^ | None | FLAG | *Sal*I | LC171736 |  |
| R4pDD613-MD8 | pUC119 | Amp^r^, Cm^r^ | None | 3xHA | *Sal*I | LC171737 |  |
| R4pDD616-MD8 | pUC119 | Amp^r^, Cm^r^ | None | 4xMyc | *Sal*I | LC171738 |  |
| R4pDD619-MD8 | pUC119 | Amp^r^, Cm^r^ | None | 10xMyc | *Sal*I | LC171739 |  |
| R4pDD622-MD8 | pUC119 | Amp^r^, Cm^r^ | None | GST | *Sal*I | LC171740 |  |
| R4pDD625-MD8 | pUC119 | Amp^r^, Cm^r^ | None | T7 | *Sal*I | LC171741 |  |
| R4pDD633-MD8 | pUC119 | Amp^r^, Cm^r^ | None | GUS | *Sal*I | LC171742 |  |
| R4pDD635-MD8 | pUC119 | Amp^r^, Cm^r^ | None | LUC | *Sal*I | LC171743 |  |
| R4pDD640-MD8 | pUC119 | Amp^r^, Cm^r^ | None | EYFP | *Sal*I | LC171744 |  |
| R4pDD643-MD8 | pUC119 | Amp^r^, Cm^r^ | None | ECFP | *Sal*I | LC171745 |  |
| R4pDD650-MD8 | pUC119 | Amp^r^, Cm^r^ | None | G3GFP | *Sal*I | LC171746 |  |
| R4pDD653-MD8 | pUC119 | Amp^r^, Cm^r^ | None | mRFP | *Sal*I | LC171747 |  |
| R4pDD659-MD8 | pUC119 | Amp^r^, Cm^r^ | None | TagRFP | *Sal*I | LC171748 |  |
| R4pDD600-MD8-NY2 | pUC119 | Amp^r^, Cm^r^ | None | nYFP | *Sal*I | LC171749 |  |
| R4pDD600-MD8-CY2 | pUC119 | Amp^r^, Cm^r^ | None | cYFP | *Sal*I | LC171750 |  |
| R4pGWB6401-MD8 | pPZP | Spc^r^, Cm^r^ | Pnos:NPTII (Km^r^) | None | *Sal*I or *Xho*I | LC171751 |  |
| R4pGWB6404-MD8 | pPZP | Spc^r^, Cm^r^ | Pnos:NPTII (Km^r^) | sGFP | *Sal*I or *Xho*I | LC171752 |  |
| R4pGWB6407-MD8 | pPZP | Spc^r^, Cm^r^ | Pnos:NPTII (Km^r^) | 6xHis | *Sal*I or *Xho*I | LC171753 |  |
| R4pGWB6410-MD8 | pPZP | Spc^r^, Cm^r^ | Pnos:NPTII (Km^r^) | FLAG | *Sal*I or *Xho*I | LC171754 |  |
| R4pGWB6413-MD8 | pPZP | Spc^r^, Cm^r^ | Pnos:NPTII (Km^r^) | 3xHA | *Sal*I or *Xho*I | LC171755 |  |
| R4pGWB6416-MD8 | pPZP | Spc^r^, Cm^r^ | Pnos:NPTII (Km^r^) | 4xMyc | *Sal*I or *Xho*I | LC171756 |  |
| R4pGWB6419-MD8 | pPZP | Spc^r^, Cm^r^ | Pnos:NPTII (Km^r^) | 10xMyc | *Sal*I or *Xho*I | LC171757 |  |
| R4pGWB6422-MD8 | pPZP | Spc^r^, Cm^r^ | Pnos:NPTII (Km^r^) | GST | *Sal*I or *Xho*I | LC171758 |  |
| R4pGWB6425-MD8 | pPZP | Spc^r^, Cm^r^ | Pnos:NPTII (Km^r^) | T7 | *Sal*I or *Xho*I | LC171759 |  |
| R4pGWB6433-MD8 | pPZP | Spc^r^, Cm^r^ | Pnos:NPTII (Km^r^) | GUS | *Sal*I or *Xho*I | LC171760 |  |
| R4pGWB6435-MD8 | pPZP | Spc^r^, Cm^r^ | Pnos:NPTII (Km^r^) | LUC | *Sal*I or *Xho*I | LC171761 |  |
| R4pGWB6440-MD8 | pPZP | Spc^r^, Cm^r^ | Pnos:NPTII (Km^r^) | EYFP | *Sal*I or *Xho*I | LC171762 |  |
| R4pGWB6443-MD8 | pPZP | Spc^r^, Cm^r^ | Pnos:NPTII (Km^r^) | ECFP | *Sal*I or *Xho*I | LC171763 |  |
| R4pGWB6450-MD8 | pPZP | Spc^r^, Cm^r^ | Pnos:NPTII (Km^r^) | G3GFP | *Sal*I or *Xho*I | LC171764 |  |
| R4pGWB6453-MD8 | pPZP | Spc^r^, Cm^r^ | Pnos:NPTII (Km^r^) | mRFP | *Sal*I or *Xho*I | LC171765 |  |
| R4pGWB6459-MD8 | pPZP | Spc^r^, Cm^r^ | Pnos:NPTII (Km^r^) | TagRFP | *Sal*I or *Xho*I | LC171766 |  |
| R4pGWB6400-MD8-NY2 | pPZP | Spc^r^, Cm^r^ | Pnos:NPTII (Km^r^) | nYFP | *Sal*I or *Xho*I | LC171767 |  |
| R4pGWB6400-MD8-CY2 | pPZP | Spc^r^, Cm^r^ | Pnos:NPTII (Km^r^) | cYFP | *Sal*I or *Xho*I | LC171768 |  |
| R4pGWB6501-MD8 | pPZP | Spc^r^, Cm^r^ | Pnos:HPT (Hyg^r^) | None | *Sal*I or *Xho*I | LC171769 |  |
| R4pGWB6504-MD8 | pPZP | Spc^r^, Cm^r^ | Pnos:HPT (Hyg^r^) | sGFP | *Sal*I or *Xho*I | LC171770 |  |
| R4pGWB6507-MD8 | pPZP | Spc^r^, Cm^r^ | Pnos:HPT (Hyg^r^) | 6xHis | *Sal*I or *Xho*I | LC171771 |  |
| R4pGWB6510-MD8 | pPZP | Spc^r^, Cm^r^ | Pnos:HPT (Hyg^r^) | FLAG | *Sal*I or *Xho*I | LC171772 |  |
| R4pGWB6513-MD8 | pPZP | Spc^r^, Cm^r^ | Pnos:HPT (Hyg^r^) | 3xHA | *Sal*I or *Xho*I | LC171773 |  |
| R4pGWB6516-MD8 | pPZP | Spc^r^, Cm^r^ | Pnos:HPT (Hyg^r^) | 4xMyc | *Sal*I or *Xho*I | LC171774 |  |
| R4pGWB6519-MD8 | pPZP | Spc^r^, Cm^r^ | Pnos:HPT (Hyg^r^) | 10xMyc | *Sal*I or *Xho*I | LC171775 |  |
| R4pGWB6522-MD8 | pPZP | Spc^r^, Cm^r^ | Pnos:HPT (Hyg^r^) | GST | *Sal*I or *Xho*I | LC171776 |  |
| R4pGWB6525-MD8 | pPZP | Spc^r^, Cm^r^ | Pnos:HPT (Hyg^r^) | T7 | *Sal*I or *Xho*I | LC171777 |  |
| R4pGWB6533-MD8 | pPZP | Spc^r^, Cm^r^ | Pnos:HPT (Hyg^r^) | GUS | *Sal*I or *Xho*I | LC171778 |  |
| R4pGWB6535-MD8 | pPZP | Spc^r^, Cm^r^ | Pnos:HPT (Hyg^r^) | LUC | *Sal*I or *Xho*I | LC171779 |  |
| R4pGWB6540-MD8 | pPZP | Spc^r^, Cm^r^ | Pnos:HPT (Hyg^r^) | EYFP | *Sal*I or *Xho*I | LC171780 |  |
| R4pGWB6543-MD8 | pPZP | Spc^r^, Cm^r^ | Pnos:HPT (Hyg^r^) | ECFP | *Sal*I or *Xho*I | LC171781 |  |
| R4pGWB6550-MD8 | pPZP | Spc^r^, Cm^r^ | Pnos:HPT (Hyg^r^) | G3GFP | *Sal*I or *Xho*I | LC171782 |  |
| R4pGWB6553-MD8 | pPZP | Spc^r^, Cm^r^ | Pnos:HPT (Hyg^r^) | mRFP | *Sal*I or *Xho*I | LC171783 |  |
| R4pGWB6559-MD8 | pPZP | Spc^r^, Cm^r^ | Pnos:HPT (Hyg^r^) | TagRFP | *Sal*I or *Xho*I | LC171784 |  |
| R4pGWB6500-MD8-NY2 | pPZP | Spc^r^, Cm^r^ | Pnos:HPT (Hyg^r^) | nYFP | *Sal*I or *Xho*I | LC171785 |  |
| R4pGWB6500-MD8-CY2 | pPZP | Spc^r^, Cm^r^ | Pnos:HPT (Hyg^r^) | cYFP | *Sal*I or *Xho*I | LC171786 |  |
| R4pGWB6601-MD8 | pPZP | Spc^r^, Cm^r^ | Pnos:bar (BASTA^r^) | None | *Xho*I | LC171787 |  |
| R4pGWB6604-MD8 | pPZP | Spc^r^, Cm^r^ | Pnos:bar (BASTA^r^) | sGFP | *Xho*I | LC171788 |  |
| R4pGWB6607-MD8 | pPZP | Spc^r^, Cm^r^ | Pnos:bar (BASTA^r^) | 6xHis | *Xho*I | LC171789 |  |
| R4pGWB6610-MD8 | pPZP | Spc^r^, Cm^r^ | Pnos:bar (BASTA^r^) | FLAG | *Xho*I | LC171790 |  |
| R4pGWB6613-MD8 | pPZP | Spc^r^, Cm^r^ | Pnos:bar (BASTA^r^) | 3xHA | *Xho*I | LC171791 |  |
| R4pGWB6616-MD8 | pPZP | Spc^r^, Cm^r^ | Pnos:bar (BASTA^r^) | 4xMyc | *Xho*I | LC171792 |  |
| R4pGWB6619-MD8 | pPZP | Spc^r^, Cm^r^ | Pnos:bar (BASTA^r^) | 10xMyc | *Xho*I | LC171793 |  |
| R4pGWB6622-MD8 | pPZP | Spc^r^, Cm^r^ | Pnos:bar (BASTA^r^) | GST | *Xho*I | LC171794 |  |
| R4pGWB6625-MD8 | pPZP | Spc^r^, Cm^r^ | Pnos:bar (BASTA^r^) | T7 | *Xho*I | LC171795 |  |
| R4pGWB6633-MD8 | pPZP | Spc^r^, Cm^r^ | Pnos:bar (BASTA^r^) | GUS | *Xho*I | LC171796 |  |
| R4pGWB6635-MD8 | pPZP | Spc^r^, Cm^r^ | Pnos:bar (BASTA^r^) | LUC | *Xho*I | LC171797 |  |
| R4pGWB6640-MD8 | pPZP | Spc^r^, Cm^r^ | Pnos:bar (BASTA^r^) | EYFP | *Xho*I | LC171798 |  |
| R4pGWB6643-MD8 | pPZP | Spc^r^, Cm^r^ | Pnos:bar (BASTA^r^) | ECFP | *Xho*I | LC171799 |  |
| R4pGWB6650-MD8 | pPZP | Spc^r^, Cm^r^ | Pnos:bar (BASTA^r^) | G3GFP | *Xho*I | LC171800 |  |
| R4pGWB6653-MD8 | pPZP | Spc^r^, Cm^r^ | Pnos:bar (BASTA^r^) | mRFP | *Xho*I | LC171801 |  |
| R4pGWB6659-MD8 | pPZP | Spc^r^, Cm^r^ | Pnos:bar (BASTA^r^) | TagRFP | *Xho*I | LC171802 |  |
| R4pGWB6600-MD8-NY2 | pPZP | Spc^r^, Cm^r^ | Pnos:bar (BASTA^r^) | nYFP | *Xho*I | LC171803 |  |
| R4pGWB6600-MD8-CY2 | pPZP | Spc^r^, Cm^r^ | Pnos:bar (BASTA^r^) | cYFP | *Xho*I | LC171804 |  |
| R4pGWB6701-MD8 | pPZP | Spc^r^, Cm^r^ | Pnos:GPT (Tunica^r^) | None | *Sal*I | LC171805 |  |
| R4pGWB6704-MD8 | pPZP | Spc^r^, Cm^r^ | Pnos:GPT (Tunica^r^) | sGFP | *Sal*I | LC171806 |  |
| R4pGWB6707-MD8 | pPZP | Spc^r^, Cm^r^ | Pnos:GPT (Tunica^r^) | 6xHis | *Sal*I | LC171807 |  |
| R4pGWB6710-MD8 | pPZP | Spc^r^, Cm^r^ | Pnos:GPT (Tunica^r^) | FLAG | *Sal*I | LC171808 |  |
| R4pGWB6713-MD8 | pPZP | Spc^r^, Cm^r^ | Pnos:GPT (Tunica^r^) | 3xHA | *Sal*I | LC171809 |  |
| R4pGWB6716-MD8 | pPZP | Spc^r^, Cm^r^ | Pnos:GPT (Tunica^r^) | 4xMyc | *Sal*I | LC171810 |  |
| R4pGWB6719-MD8 | pPZP | Spc^r^, Cm^r^ | Pnos:GPT (Tunica^r^) | 10xMyc | *Sal*I | LC171811 |  |
| R4pGWB6722-MD8 | pPZP | Spc^r^, Cm^r^ | Pnos:GPT (Tunica^r^) | GST | *Sal*I | LC171812 |  |
| R4pGWB6725-MD8 | pPZP | Spc^r^, Cm^r^ | Pnos:GPT (Tunica^r^) | T7 | *Sal*I | LC171813 |  |
| R4pGWB6733-MD8 | pPZP | Spc^r^, Cm^r^ | Pnos:GPT (Tunica^r^) | GUS | *Sal*I | LC171814 |  |
| R4pGWB6735-MD8 | pPZP | Spc^r^, Cm^r^ | Pnos:GPT (Tunica^r^) | LUC | *Sal*I | LC171815 |  |
| R4pGWB6740-MD8 | pPZP | Spc^r^, Cm^r^ | Pnos:GPT (Tunica^r^) | EYFP | *Sal*I | LC171816 |  |
| R4pGWB6743-MD8 | pPZP | Spc^r^, Cm^r^ | Pnos:GPT (Tunica^r^) | ECFP | *Sal*I | LC171817 |  |
| R4pGWB6750-MD8 | pPZP | Spc^r^, Cm^r^ | Pnos:GPT (Tunica^r^) | G3GFP | *Sal*I | LC171818 |  |
| R4pGWB6753-MD8 | pPZP | Spc^r^, Cm^r^ | Pnos:GPT (Tunica^r^) | mRFP | *Sal*I | LC171819 |  |
| R4pGWB6759-MD8 | pPZP | Spc^r^, Cm^r^ | Pnos:GPT (Tunica^r^) | TagRFP | *Sal*I | LC171820 |  |
| R4pGWB6700-MD8-NY2 | pPZP | Spc^r^, Cm^r^ | Pnos:GPT (Tunica^r^) | nYFP | *Sal*I | LC171821 |  |
| R4pGWB6700-MD8-CY2 | pPZP | Spc^r^, Cm^r^ | Pnos:GPT (Tunica^r^) | cYFP | *Sal*I | LC171822 |  |
