# Supplementary material for: Development of an R4 dual-site (R4DS) gateway cloning system enabling the efficient simultaneous cloning of two desired sets of promoters and open reading frames in a binary vector for plant research
Source: PLoS One. 2017 May 16;12(5):e0177889. doi: 10.1371/journal.pone.0177889 (PMC5433782; doi:10.1371/journal.pone.0177889)
Supplement: S2 Table — Ten binary clones containing different combinations of Pro1:ORF1-tag1-Pro2:ORF2-tag2 were constructed. The total vector size and size of HindIII fragments are indicated in base pairs (bp). PMUTE, MUTE promoter; PSDD1, SDD1 promoter; Mt, mitochondria-targeting signal; PTS2, peroxisome-targeting signal type 2; Pt, plastid-targeting signal. (DOCX) [file pone.0177889.s003.docx]

S2 Table. Binary clones constructed in this study. Ten binary clones containing different combinations of Pro1:ORF1-tag1-Pro2:ORF2-tag2 were constructed. The total vector size and size of *Hin*dIII fragments are indicated in base pairs (bp). P_MUTE_, *MUTE* promoter; P_SDD1_, *SDD1* promoter; Mt, mitochondria-targeting signal; PTS2, peroxisome-targeting signal type 2; Pt, plastid-targeting signal.

| **Binary clone** | **Total size**  **(bp)** | ***Hin*dIII**  **Fragment 1 Fragment 2 Fragment 3 Fragment 4 Fragment 5** | | | | |
| --- | --- | --- | --- | --- | --- | --- |
| P_MUTE_:Mt-G3GFP-P_SDD1_:Mt-TagRFP | 17,972 | 2,965 | 2,123 | 135 | 965 | 11,784 |
| P_MUTE_:PTS2-G3GFP-P_SDD1_:PTS2-TagRFP | 18,008 | 2,983 | 2,123 | 135 | 965 | 11,802 |
| P_MUTE_:Pt-G3GFP-P_SDD1_:Pt-TagRFP | 18,050 | 3,004 | 2,123 | 135 | 965 | 11,823 |
| P_MUTE_:Mt-G3GFP-P_SDD1_:PTS2-TagRFP | 17,990 | 2,965 | 2,123 | 135 | 965 | 11,802 |
| P_MUTE_:Mt-G3GFP-P_SDD1_:Pt-TagRFP | 18,011 | 2,965 | 2,123 | 135 | 965 | 11,823 |
| P_MUTE_:Mt-TagRFP-P_SDD1_:Mt-G3GFP | 17,972 | 5,085 | 135 | 965 | 1,913 | 9,874 |
| P_MUTE_:PTS2-TagRFP-P_SDD1_:PTS2-G3GFP | 18,008 | 5,103 | 135 | 965 | 1,931 | 9,874 |
| P_MUTE_:Pt-TagRFP-P_SDD1_:Pt-G3GFP | 18,050 | 5,124 | 135 | 965 | 1,952 | 9,874 |
| P_MUTE_:Pt-TagRFP-P_SDD1_:PTS2-G3GFP | 18,029 | 5,124 | 135 | 965 | 1,931 | 9,874 |
| P_SDD1_:Mt- TagRFP-P_MUTE_:Mt-G3GFP | 17,972 | 135 | 965 | 4,033 | 2,965 | 9,874 |
