# Supplementary material for: Development of an R4 dual-site (R4DS) gateway cloning system enabling the efficient simultaneous cloning of two desired sets of promoters and open reading frames in a binary vector for plant research
Source: PLoS One. 2017 May 16;12(5):e0177889. doi: 10.1371/journal.pone.0177889 (PMC5433782; doi:10.1371/journal.pone.0177889)
Supplement: S3 Table — Restriction sites are underlined. (DOCX) [file pone.0177889.s004.docx]

| S3 Table. Oligonucleotides used in this study.  Restriction sites are underlined. | |
| --- | --- |
| Oligos | Sequence |
| Linker |  |
| *Swa*I | 5ʹ-AGCTGGTGATTTAAATCACC-3ʹ |
| Adaptors |  |
| *Hin*dIII*-Xba*I*-Sac*I*-Eco*RI-F | 5ʹ-AGCTAGAATTCGGAGCTCGTCTAGAGAAGCTTAC-3ʹ |
| *Hin*dIII*-Xba*I*-Sac*I*-Eco*RI-R | 5ʹ-CTAGTAAGCTTCTCTAGACGAGCTCCGAATTCT-3ʹ |
| *Pme*I-F | 5ʹ-AGCTCACTCGTTTAAACACTGC-3ʹ |
| *Pme*I-R | 5ʹ-AGCTGCAGTGTTTAAACGAGTG-3ʹ |
| *Swa*I*-Not*I-*Asc*I-*Eco*RI-F | 5ʹ-AATTAATTTAAATGCGGCCGCGGCGCGCCG-3ʹ |
| *Swa*I*-Not*I-*Asc*I-*Eco*RI-R | 5ʹ-AATTCGGCGCGCCGCGGCCGCATTTAAATT-3ʹ |
| *Swa*I-F | 5ʹ-GGCCACATTTAAATG-3ʹ |
| *Swa*I-R | 5ʹ-CATGCATTTAAATGT-3ʹ |
| *Xho*I-*Bsp*EI-F | 5'-TCGAGTCTCCCTCATT-3' |
| *Xho*I-*Bsp*EI-R | 5'- CAGAGGGAGTAAGGCC-3' |
| Primers |  |
| proximal-to-*att*L1 | 5ʹ-TCGCGTTAACGCTAGCATGGATCTC-3ʹ |
| 3’-*att*L4-F | 5ʹ-TGCCAACTTTGTATAGAAAAGTAG-3ʹ |
| 5’-*att*L4-R | 5ʹ-AGCCTACTTTTCTATACAAAGTTG-3ʹ |
| Tnos-F | 5'-AATAAAGTTTCTTAAGATTGAATCC-3' |
| Tnos-R | 5ʹ-GATCTAGTAACATAGATGAC-3ʹ |
| 3’-*att*L3-F | 5’-ACCCAACTTTATTATACAAAGTTG-3’ |
| 5’-*att*L3-R | 5'-TGCCAACTTTGTATAATAAAGTTG-3' |
| proximal-to-*att*L2 | 5’-GTAACATCAGAGATTTTGAGACAC-3’ |
| 3’-*att*L5-F | 5’-TGCCAACTTTGTATACAAAAGTAG-3’ |
| 5’-*att*L5-R | 5’-AGCCTACTTTTGTATACAAAGTTG-3’ |
| 3’-*att*L6-F | 5’-ACCCAACTTTTTAATACAAAGTTG-3’ |
| 5’-*att*L6-R | 5’-TGCCAACTTTGTATTAAAAAGTTG-3’ |
| *Not*I-*att*R4 | 5’-CTGCGGCCGCGTGGATCCCCCATCACAAC-3’ |
| *Asc*I-*att*R3 | 5’-CTGGCGCGCCAGCTTGATATCACAACTTTGTAT-3’ |
| *Not*I-*att*R5 | 5’-CTGCGGCCGCGTGGATCCCCCATCACAACTTTGTATACAAAAG-3’ |
| *Asc*I-*att*R6 | 5’-CTGGCGCGCCAGCTTGATATCACAACTTTGTATTAAAAAG-3’ |
| MD8-F | 5ʹ-TCATCACACGTAATATCATCC-3ʹ |
| MD8-*Hin*dIII-R | 5ʹ-AAGCTTAGATTTTCAGCTTTCTTTTTTTTCC-3ʹ |
| MD8-*Hin*dIII-Cm^r^ (ATG)-R | 5ʹ-GAAGCATAAAGTGTAAAGCCTAAGCTTAGATTTTCAGCTTTCTTTTTTTTCC-3ʹ |
| 5’-Cm^r^-F | 5’-TAAGAGGTTCCAACTTTCAC-3’ |
| 3’-Cm^r^-R | 5’-ACATATCAGTATATATTCTTATACC-3’ |
| Cm^r^-F | 5ʹ-AGGCTTTACACTTTATGCTTC-3ʹ |
| Cm^r^-*Hin*dIII-R | 5ʹ-AAGCTTCCTTACCAGACCGGAGATAT-3ʹ |
| 3’-Cm^r^-d*Eco*RI-F | 5’-CATCCGGAATTTCGTATGGCAA-3’ |
| 5’-Cm^r^-d*Eco*RI-R | 5’-TTGCCATACGAAATTCCGGATG-3’ |
| P_MUTE_-*att*B1 | 5'-GGGGACTGCTTTTTTGTACAAACTTGTGACACTGATACTTAATTGATCAAGATTC-3' |
| P_MUTE_-*att*B4 | 5'-GGGGACAACTTTGTATAGAAAAGTTGGCTGAGACACTCCAGCAATTTGAAAAATCC-3' |
| F_1_ATPg-*att*B1 | 5’-AAAAAGCAGGCTTTATGGCAATGGCTGTTTTCCG-3’ |
| F_1_ATPg-*att*B2 | 5’-AGAAAGCTGGGTTAGATCGAACTCCAAGAAGTCC-3’ |
| RBCS1A-*att*B1 | 5’-AAAAAGCAGGCTTTATGGCTTCCTCTATGCTCTC-3’ |
| RBCS1A-*att*B2 | 5’-AGAAAGCTGGGTTGCAGTTAACTCTTCCGCCG-3’ |
| *att*B1 adaptor | 5'-GGGGACAAGTTTGTACAAAAAAGCAGGCT -3' |
| *att*B2 adaptor | 5'-GGGGACCACTTTGTACAAGAAAGCTGGGT-3' |
