# Supplementary material for: Development of an R4 dual-site (R4DS) gateway cloning system enabling the efficient simultaneous cloning of two desired sets of promoters and open reading frames in a binary vector for plant research
Source: PLoS One. 2017 May 16;12(5):e0177889. doi: 10.1371/journal.pone.0177889 (PMC5433782; doi:10.1371/journal.pone.0177889)
Supplement: S1 Text — (DOCX) [file pone.0177889.s005.docx]

# **S1 Text:** Detailed description of plasmid construction.

A representative scheme for the construction of vectors is illustrated in S1 Fig. All oligonucleotides used as linkers, adaptors, and primers are listed in S3 Table.

## Construction of R4pGWB6x01-MD8

The 35S promoter was removed from pUGW2 [1] by digestion with *Xba*I to make pUGW2-∆35S. The chloramphenicol resistance (Cm^r^) fragment between *att*R1 and *att*R2 was removed from pUGW2-∆35S by digestion with *Bam*HI to make pUGW2-∆(35S, Cm^r^). The *Swa*I-*Not*I-*Asc*I-*Eco*RI adaptor was introduced into the *Eco*RI site of pUGW2-∆(35S, Cm^r^) to make pUGW2-∆(35S, Cm^r^)-SNAE. Destruction of the *Eco*RI site in Cm^r^ was performed as follows. The outermost upstream region of the Cm^r^ fragment was amplified using pDONR201 (Invitrogen) as a template with the primers 5’-Cm^r^-F and 5’-Cm^r^-d*Eco*RI-R. The outermost downstream region of the Cm^r^ fragment was amplified using pDONR201 as a template with 3’-Cm^r^-d*Eco*RI-F and 3’-Cm^r^-R. Products were diluted, mixed, and used in a second PCR with the primers 5’-Cm^r^-F and 3’-Cm^r^-R. The amplified modified Cm^r^ fragment was introduced into the *Swa*I site of pUGW2-∆(35S, Cm^r^)-SNAE to make pUGW2-∆35S-Cm^r^-NAE (*att*R1-*ccd*B-*att*R2-Tnos-Cm^r^). The *Sal*I site of pDONR-L1R1R2L2 [2] was destroyed by *Sal*I digestion followed by a treatment with the Klenow enzyme. The *Not*I-*Nco*I region within Gateway reading cassette A of the resulting plasmid was replaced with a *Swa*I adaptor to make pDONR-L1R1-*Swa*I-R2L2. The *Not*I-*att*R4-*Swa*I-*att*R3-*Asc*I sequence was amplified using pDONR-L1R1-*Swa*I-R2L2 as a template with the primers *Not*I-*att*R4 and *Asc*I-*att*R3. The amplified product was digested with *Not*I and *Asc*I, then introduced into the *Not*I and *Asc*I sites of pUGW2-∆35S-Cm^r^-NAE to make pUGW3001 (*att*R1-*ccd*B-*att*R2-Tnos-Cm^r^-*att*R4-*ccd*B-*att*R3). The *Not*I-*att*R5-*ccd*B-*att*R6-*Asc*I sequence was amplified using pUGW3001 as a template with the primers *Not*I-*att*R5 and *Asc*I-*att*R6. The amplified product was digested with *Not*I and *Asc*I, then *att*R4-*ccd*B-*att*R3 of pUGW3001 was replaced with *att*R5-*ccd*B-*att*R6 to make pUGW6001 (*att*R1-*ccd*B-*att*R2-Tnos-Cm^r^-*att*R5-*ccd*B-*att*R6). The *att*R1 sequence of pUGW6001 was replaced with *att*R4 prepared from R4pUGW1 [3] by *Hin*dIII and *Xho*I digestion to make R4pUGW6001 (*att*R4-*ccd*B-*att*R2-Tnos-Cm^r^-*att*R5-*ccd*B-*att*R6).

The *Swa*I linker was introduced into the *Hin*dIII site of pGWB400, pGWB500 [4], pGWB600 [5], and pGWB700 [6] to make pGWB400-*Swa*I, pGWB500-*Swa*I, pGWB600-*Swa*I, and pGWB700-*Swa*I, respectively. A recombinant (Fusion) PCR technique was used to link the MD8 sequence [7] with the Cm^r^ sequence. The MD8 fragment was amplified by PCR using *Arabidopsis thaliana* genomic DNA as a template with the MD8-F and MD8-*Hin*dIII-Cm^r^ (ATG)**-**R primers. The Cm^r^ sequence was amplified by PCR using pGWB401 [4] as a template with the Cm^r^-F and Cm^r^-*Hin*dIII**-**R primers**.** These products were diluted, mixed, and subjected to a second PCR with the MD8-F and Cm^r^-*Hin*dIII**-**R primers to make the MD8-*Hin*dIII-Cm^r^-*Hin*dIII fragment, and then introduced into the *Swa*I site of pGWB400-*Swa*I, pGWB500-*Swa*I, pGWB600-*Swa*I, and pGWB700-*Swa*I to make pGWB400-MD8-*Hin*dIII-Cm^r^-*Hin*dIII, pGWB500-MD8-*Hin*dIII-Cm^r^-*Hin*dIII, pGWB600-MD8-*Hin*dIII-Cm^r^-*Hin*dIII, and pGWB700-MD8-*Hin*dIII-Cm^r^-*Hin*dIII, respectively. The *Hin*dIII-Cm^r^-*Hin*dIII fragment was excised by *Hin*dIII digestion followed by self-ligation to make pGWB400-MD8, pGWB500-MD8, pGWB600-MD8, and pGWB700-MD8, respectively (MD8-*Hin*dIII-*Xba*I-*Sac*I-Tnos). The *att*R4-*ccd*B-*att*R2-Tnos-Cm^r^-*att*R5-*ccd*B-*att*R6 sequence of R4pUGW6001 was introduced into the *Hin*dIII-*Asc*I sites of pGWB400-MD8, pGWB500-MD8, pGWB600-MD8, and pGWB700-MD8 to generate R4pGWB6401-MD8, R4pGWB6501-MD8, R4pGWB6601-MD8, and R4pGWB6701-MD8, respectively (MD8-*att*R4-*ccd*B-*att*R2-Tnos-Cm^r^-*att*R5-*ccd*B-*att*R6).

## Construction of R4pGWB6xxx-MD8 for fusion with a tag

The 35S promoter was prepared from pGWB401 [4] and introduced into the *Hin*dIII-*Xba*I site of pUGW3001 to make pUGW3002. The Cm^r^ of pUGWxx [1], pGWnY, and pGWcY [8] was removed by digestion with *Bam*HI to make pUGWxx-∆Cm^r^, pGWnY-∆Cm^r^, and pGWcY-∆Cm^r^, respectively. The *Xba*I-*Sac*I fragment containing the *att*R1-*ccd*B-*att*R2-tag was prepared from pUGWxx-∆Cm^r^, pGWnY-∆Cm^r^, and pGWcY-∆Cm^r^, and introduced into pUGW3002 to make pUGW30xx, pUGW3000-nY, and pUGW3000-cY, respectively.

### Construction of R4pGWB64xx-MD8 (kanamycin resistance) and R4pGWB66xx-MD8 (BASTA resistance)

The *Xho*I-*Bsp*EI adaptor was introduced into R4pGWB6401-MD8 and R4pGWB6601-MD8 to make R4pGWB6401-MD8/*Xho*I-*Bsp*EI and R4pGWB6601-MD8/*Xho*I-*Bsp*EI, respectively. The *Xho*I-*ccd*B-*att*R2-tag-Tnos-Cm^r^-*Bsp*EI fragment was prepared from pUGW30xx, pUGW3000-nY, and pUGW3000-cY and inserted into R4pGWB6401-MD8/*Xho*I-*Bsp*EI to make R4pGWB6404-MD8 to R4pGWB6459-MD8, R4pGWB6400-MD8-NY2, and R4pGWB6400-MD8-CY2, respectively. Similarly, R4pGWB6604-MD8 to R4pGWB6659-MD8, R4pGWB6600-MD8-NY2, and R4pGWB6600-MD8-CY2 were generated by replacing R4pGWB6401-MD8/*Xho*I-*Bsp*EI with R4pGWB6601-MD8/*Xho*I-*Bsp*EI. In LUC containing the *Bsp*EI site in its sequence, *Xho*I-*ccd*B-*att*R2-mRFP-*Sac*I of R4pGWB6454-MD8 and R4pGWB6654-MD8 was replaced with *Xho*I-*ccd*B-*att*R2-LUC-*Sac*I obtained from pUGW3035. The resulting plasmids were designated R4pGWB6435-MD8 and R4pGWB6635-MD8, respectively.

### Construction of R4pGWB65xx-MD8 (hygromycin resistance)

The *Hin*dIII-*att*R4-*ccd*B-*att*R2-tag-Tnos-Cm^r^-*att*R5-*ccd*B-*att*R6-*Asc*I fragment was prepared from R4pGWB64xx-MD8 and R4pGWB6400-MD8-NY2 vectors and inserted into pGWB500-MD8 by *Hin*dIII and *Asc*I digestion. The resulting plasmids were designated R4pGWB65xx-MD8 and R4pGWB6500-MD8-NY2. Regarding LUC, G3GFP, mRFP, and cYFP having the *Hin*dIII site at their 3’ terminals, *Xho*I-*ccd*B-*att*R2-EYFP-*Sac*I of R4pGWB6540-MD8 was replaced with *Xho*I-*ccd*B-*att*R2-LUC-*Sac*I (from R4pGWB6435-MD8), *Xho*I-*ccd*B-*att*R2-G3GFP-*Sac*I (from R4pGWB6450-MD8), *Xho*I-*ccd*B-*att*R2-mRFP-*Sac*I (from R4pGWB6453-MD8), and *Xho*I-*ccd*B-*att*R2-cYFP-*Sac*I (from R4pGWB6400-MD8-CY2) to make R4pGWB6535-MD8, R4pGWB6550-MD8, R4pGWB6553-MD8, and R4pGWB6500-MD8-CY2, respectively.

### Construction of R4pGWB67xx-MD8 (tunicamycin resistance)

The *Hin*dIII-*att*R4-*ccd*B-*att*R2-tag-Tnos-Cm^r^-*att*R5-*ccd*B-*att*R6-*Asc*I fragment was prepared from the R4pGWB64xx-MD8 and R4pGWB6400-MD8-NY2 vectors and inserted into pGWB700-MD8 by *Hin*dIII and *Asc*I digestion. The resulting plasmids were designated R4pGWB67xx-MD8 and R4pGWB6700-MD8-NY2. Regarding LUC, G3GFP, mRFP, and cYFP containing the *Hin*dIII site at their 3’ terminals, *Asc*I-(NPTII marker)-LB-*Age*I of R4pGWBB6435-MD8, R4pGWB6450-MD8, R4pGWB6453-MD8, and R4pGWB6400-MD8-CY2 was replaced with *Asc*I-(GPT marker)-LB-*Age*I from pGWB700-MD8 to make R4pGWB6735-MD8, R4pGWB6750-MD8, R4pGWB6753-MD8, and R4pGWB6700-MD8-CY2 respectively.

## Construction of R4pDD6xx-MD8

The *Hin*dIII-*Xba*I-*Sac*I-*Eco*RI adaptor was introduced into the *Xba*I and *Hin*dIII sites between the *att*L1 and *att*L2 sites of the *A. thaliana* *BAGEL7* (AT2g43990) entry clone constructed on pDONR201 [2] to make pDONR-L1-(HXSE)-L2. The *Sac*I-*Eco*RI fragment containing the nopaline synthase terminator (Tnos) was prepared from pBI221 (Clontech) and introduced into pDONR-L1-(HXSE)-L2 to make pDONR-L1-(HX)-Tnos-L2. The *att*L4 sequence was prepared from the *att*L1 sequence as follows. The outermost upstream region of the *att*L4 sequence was amplified using pDONR-L1-(HX)-Tnos-L2 as a template with the primers proximal-to-*att*L1 and 5’-*att*L4-R. The outermost downstream region of the *att*L4 sequences was amplified using pDONR-L1-(HX)-Tnos-L2 as a template with the primers 3’-*att*L4-F and Tnos-R. The products were diluted, mixed, and used in a second PCR with the primers proximal-to-*att*L1 and Tnos-R. The amplified product was digested with *Hpa*I and *Hin*dIII, and the *att*L1 site of pDONR-L1-(HX)-Tnos-L2 was then replaced with *att*L4 to make pDONR-L4-(HX)-Tnos-L2. Similarly, the *att*L3 sequence was prepared from the *att*L2 sequence of pDONR-L1-(HX)-Tnos-L2 by using the Tnos-F, 5’-*att*L3-R, 3’-*att*L3-F, and proximal-to-*att*L2 primers in the first PCR and Tnos-F and the proximal-to-*att*L2 primers in the second PCR. The amplified product was digested with *Eco*RI and *Pvu*II, and the *att*L2 site of pDONR-L4-(HX)-Tnos-L2 was then replaced with *att*L3 to make L4L3pDD500 (*att*L4-*Hin*dIII-*Xba*I-*Sac*I-Tnos-*Eco*RI-*att*L3). Similarly, the *att*L5 sequence was prepared from the *att*L4 sequence of L4L3pDD500 by using the primers proximal-to-*att*L1, 5’-*att*L5-R, 3’-*att*L5-F, and Tnos-R in the first PCR and the primers proximal-to-*att*L1 and Tnos-R in the second PCR. The amplified product was digested with *Hpa*I and *Hin*dIII, and the *att*L4 site of L4L3pDD500 was then replaced with *att*L5 to make L5L3pDD500. The *att*L6 sequence was prepared from the *att*L3 sequence of L5L3pDD500 by using the primers Tnos-F, 5’-*att*L6-R, 3’-*att*L6-F, and proximal-to-*att*L2 in the first PCR and the primers Tnos-F and proximal-to-*att*L2 in the second PCR. The amplified product was digested with *Eco*RI and *Pvu*II, and the *att*L3 site of L5L3pDD500 was then replaced with *att*L6 to make L5L6pDD500 (*att*L5-*Hin*dIII-*Xba*I-*Sac*I-Tnos-*Eco*RI-*att*L6). The *Pme*I adaptor was introduced into the *Hin*dIII site of L5L6pDD500 to make L5L6pDD500-*Pme*I. The MD8 sequence [7] was amplified using *A. thaliana* genomic DNA as a template with the primers MD8-F and MD8-*Hin*dIII-R, and then introduced into *Pme*I site of L5L6pDD500-*Pme*I to make L5L6pDD500-MD8 (*att*L5-MD8-*Hin*dIII-*Xba*I-*Sac*I-Tnos-*Eco*RI-*att*L6). The *Hpa*I and *Nru*I fragment of L5L6PDD500-MD8, containing *att*L5-MD8-Tnos-*att*L6, was introduced into the *Sma*I site of pUC119 (TAKARA BIO, Otsu, Japan) to make pDD600-MD8. The *att*R4-Cm^r^-*ccd*B-*att*R2-tag sequences prepared from R4pGWB401 to R4pGWB459 [3] were introduced into the *Hin*dIII-*Sac*I sites of pDD600-MD8 to generate R4pDD601-MD8 to R4pDD659-MD8. Regarding LUC, G3GFP, and mRFP having the *Hin*dIII site in their sequences, the *Sal*I-*att*R2-tag-*Sac*I fragment was prepared from the pUGW35, pUGW51, and pUGW54 [1, 4] vectors and inserted into R4pDD601-MD8 to make R4pDD635-MD8, R4pDD650-MD8, and R4pDD653-MD8, respectively. Regarding nYFP and cYFP, the *Xho*I-*att*R2-tag-*Sac*I fragment was prepared from the pGWnY and pGWcY [8] vectors and inserted into R4pDD601-MD8 to make R4pDD600-MD8-NY2 and R4pDD600-MD8-CY2, respectively.

# References

1. Nakagawa T, Kurose T, Hino T, Tanaka K, Kawamukai M, Niwa Y, et al. Development of series of gateway binary vectors, pGWBs, for realizing efficient construction of fusion genes for plant transformation. J Biosci Bioeng. 2007; 104:34-41. doi: 10.1263/jbb.104.34 PMID: 17697981

2. Kimura T, Nakao A, Murata S, Kobayashi Y, Tanaka Y, Shibahara K, et al. Development of the gateway recycling cloning system for multiple linking of expression cassettes in a defined order, and direction on gateway compatible binary vectors. Biosci Biotechnol Biochem. 2013; 77:430-4. doi: 10.1271/bbb.120877 PMID: 23391940

3. Nakagawa T, Nakamura S, Tanaka K, Kawamukai M, Suzuki T, Nakamura K, et al. Development of R4 gateway binary vectors (R4pGWB) enabling high-throughput promoter swapping for plant research. Biosci Biotechnol Biochem. 2008; 72:624-9. doi: 10.1271/bbb.70678 PMID: 18256458

4. Nakagawa T, Suzuki T, Murata S, Nakamura S, Hino T, Maeo K, et al. Improved gateway binary vectors: high-performance vectors for creation of fusion constructs in transgenic analysis of plants. Biosci Biotechnol Biochem. 2007; 71:2095-100. doi: 10.1271/bbb.70216 PMID: 17690442

5. Nakamura S, Mano S, Tanaka Y, Ohnishi M, Nakamori C, Araki M, et al. Gateway binary vectors with the bialaphos resistance gene, bar, as a selection marker for plant transformation. Biosci Biotechnol Biochem. 2010; 74:1315-9. doi: 10.1271/bbb.100184 PMID: 20530878

6. Tanaka Y, Nakamura S, Kawamukai M, Koizumi N, Nakagawa T. Development of a series of gateway binary vectors possessing a tunicamycin resistance gene as a marker for the transformation of Arabidopsis thaliana. Biosci Biotechnol Biochem. 2011; 75:804-7. doi: 10.1271/bbb.110063 PMID: 21512216

7. Tachiki K, Kodama Y, Nakayama H, Shinmyo A. Determination of the in vivo distribution of nuclear matrix attachment regions using a polymerase chain reaction-based assay in Arabidopsis thaliana. J Biosci Bioeng. 2009; 108:11-9. doi: 10.1016/j.jbiosc.2009.02.010 PMID: 19577185

8. Hino T, Tanaka Y, Kawamukai M, Nishimura K, Mano S, Nakagawa T. Two Sec13p homologs, AtSec13A and AtSec13B, redundantly contribute to the formation of COPII transport vesicles in Arabidopsis thaliana. Biosci Biotechnol Biochem. 2011; 75:1848-52. doi: 10.1271/bbb.110331 PMID: 21897010
